# Analysis of Lung Adenocarcinoma Subtypes Based on Immune Signatures Identifies Clinical Implications for Cancer Therapy

**DOI:** 10.1016/j.omto.2020.03.021

**Published:** 2020-04-07

**Authors:** Feng Xu, Jie-xin Chen, Xiong-bin Yang, Xin-bin Hong, Zi-xiong Li, Ling Lin, Yong-song Chen

**Affiliations:** 1 Department of Respiratory Medicine, The First Affiliated Hospital of Shantou University Medical College, No. 57 Changping Road, Shantou, Guangdong 515041, P.R. China; 2 Department of Endocrinology, The First Affiliated Hospital of Shantou University Medical College, No. 57 Changping Road, Shantou, Guangdong 515041, P.R. China; 3 Department of Rheumatology, The First Affiliated Hospital of Shantou University Medical College, No. 57 Changping Road, Shantou, Guangdong 515041, P.R. China

**Keywords:** immune signatures, immune response, lung adenocarcinoma

## Abstract

Lung cancer is the most common cause of cancer deaths worldwide, and lung adenocarcinoma (LUAD) is the most common histological subtype. However, the prognostic and predictive outcomes differ because of this cancer type heterogeneity. LUAD subtypes were identified on the basis of the immunogenomic profiling of 29 immune signatures. We named three LUAD subtypes: Immunity High, Immunity Medium, and Immunity Low. The Immunity High subtype was characterized by immune activation, e.g., increased immune scores, elevated stromal scores and the highest infiltration of CD8^+^ T cells, and decreased tumor purities. Activated expressions of human leukocyte antigen (HLA) genes, immune checkpoint molecules, and T helper 1 (Th1)/interferon-gamma (IFNγ) gene signature were also observed in the Immunity High subtype. *N*^6^-methyladenosine (m^6^A) RNA methylation, associated with cancer initiation and progression, was reduced in the Immunity High subtype. Functional and signaling pathway enrichment analysis further showed that differentially expressed genes between the Immunity High subtype and the other subtypes mainly participated in immune response and some cancer-associated pathways. In addition, the Immunity High subtype exhibited more sensitivity to immunotherapy and chemotherapy. Finally, candidate compounds that aimed at LUAD subtype differentiation were identified. Comprehensively characterizing the LUAD subtypes based on immune signatures may help to provide potential strategies for LUAD treatment.

## Teaser

LUAD cases were classified into distinct subtypes based on immunogenomic profiling. The identification of immune signature-associated LUAD subtypes may facilitate the optimal selection for clinical application in LUAD patient treatment.

## Introduction

Lung cancer is the leading cause of cancer-associated mortality worldwide.^1^^,^^2^ Although great progress has been made toward the prevention, diagnosis, and treatment of cancer via specific cellular targets, the clinical outcome is still unsatisfactory. An increasing body of evidence reports that malignant phenotypes are influenced by a tumor-related microenvironment.^3^^,^^4^ Lung cancer, an immune-sensitive malignancy, is infiltrated by different immune cell types.^5^ Recently, cancer immunotherapy has become involved in treating all forms of cancer and has changed the landscape of cancer care. For example, inhibition of the programmed cell death 1 (PCDC1/PD-1)/CD274 molecule (CD274/PD-L1) immune checkpoint using antibodies against PD-1 rescues effector T cell function, which permits T cells to maintain their tumor cell-killing function.^6^ Moreover, in patients with high expression of PD-L1, antibodies against PD-1 are effective in treating a variety of cancers and improving overall survival.^7^^,^^8^ However, currently, cancer immunotherapy displays beneficial effects in less than 20% of patients.^9^ This may suggest that not all cancer patients could respond to immunotherapy. Lung adenocarcinoma (LUAD) is one of the major types of lung cancer, and a recent study identified an immunogenic tumor microenvironment state in non-small cell lung cancer (NSCLC) that was mainly enriched for the LUAD subtype.^10^ Also, many studies identified distinct subtypes of LUAD featured by different immune-infiltrating signatures and molecular mechanisms.^11^^,^^12^ The 5-year overall survival rate of LUAD remains at a low level of 15.9%.^13^ Therefore, it is essential to identify the LUAD subtypes based on immune signature.

In the present study, we classified LUAD into three distinct subtypes based on immunogenomic profiling: Immunity Low, Immunity Medium, and Immunity High. Furthermore, our analyses apply a new approach of identifying the optimal selection of LUAD patients responsive to immunotherapy and chemotherapy, and may provide a predictive factor for clinical application in LUAD patient treatment. Finally, recent pharmacology research has revealed the necessity to design compounds that act on multiple genes or molecular pathways.^14^, ^15^, ^16^, ^17^ In our study, we identified compounds targeting the differentiation of LUAD phenotypes, which may provide therapeutic targets for further analysis.

## Results

### Identification of LUAD Subtypes Based on Immunogenomic Profiling

**Figure 1:**
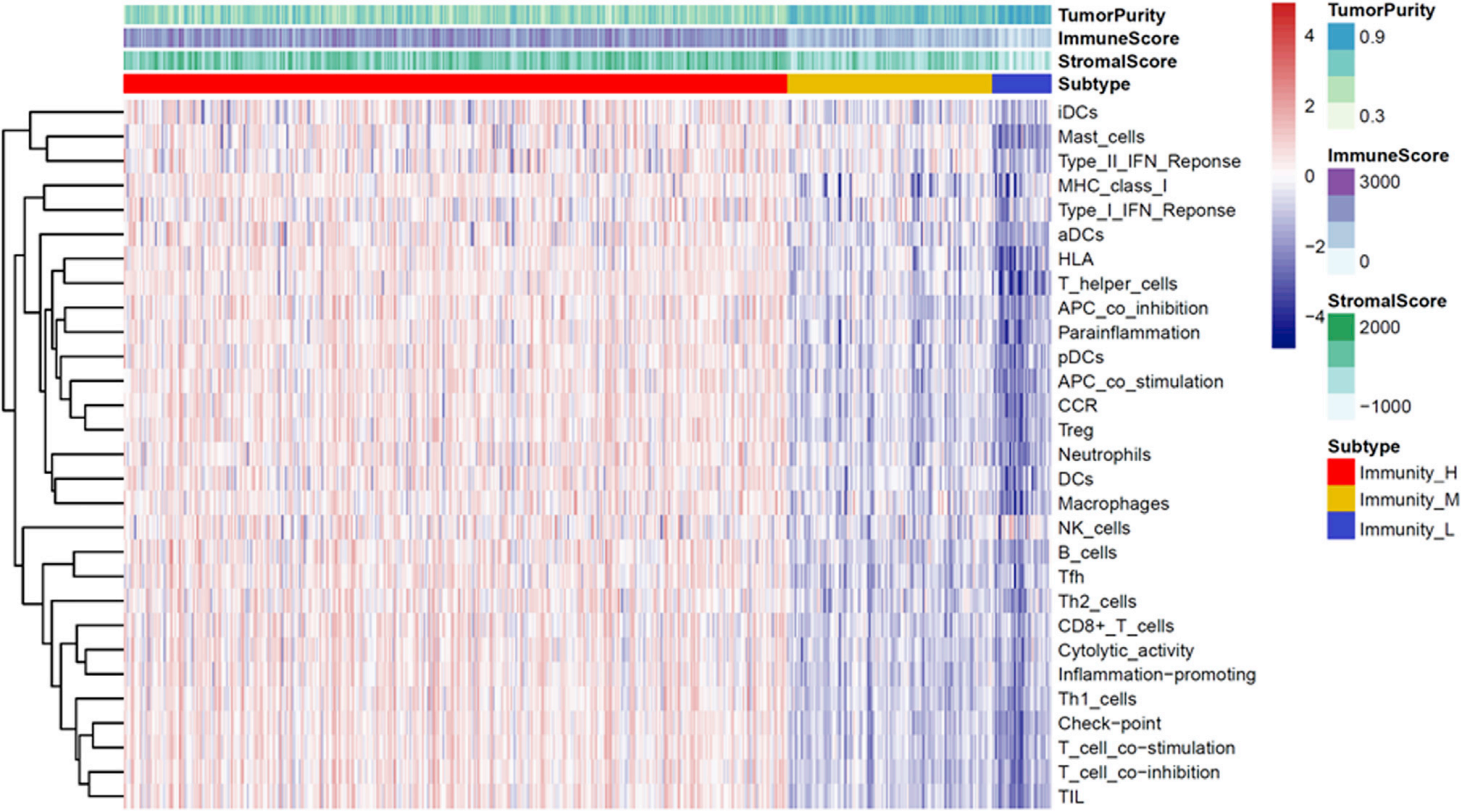
Hierarchical Clustering Yields Three Subtypes in TCGA Dataset Immunity_H, Immunity High; Immunity_L, Immunity Low; Immunity_M, Immunity Medium; LUAD, lung adenocarcinoma.

**Figure 2:**
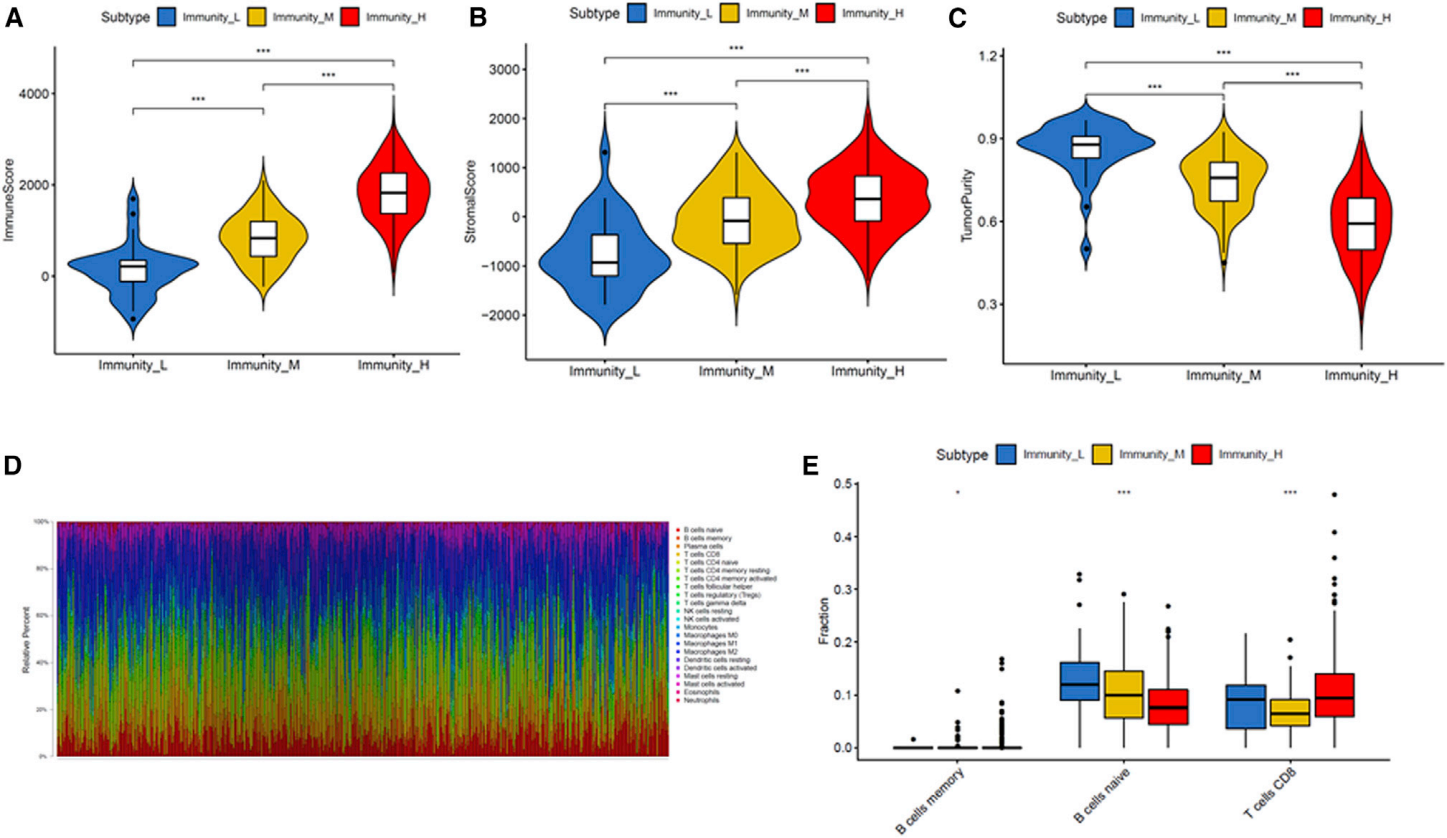
The Landscape of Immune Infiltration in LUAD Subtypes (A) Immune scores in LUAD subtypes. (B) Stromal scores in LUAD subtypes. (C) Tumor purities in LUAD subtypes. (D) Relative proportion of immune infiltration in LUAD subtypes. (E) The difference of immune cell infiltration abundance in LUAD subtypes.

To characterize the immune subtypes and immune response to cancer in LUAD patients, we analyzed the single-sample gene set enrichment analysis (ssGSEA) score using 29 immune-associated gene sets across the landscape of LUAD samples. Subsequent hierarchical cluster analysis identified characteristic immunoncological signatures, which were then used to cluster LUAD tumor types into immune subtypes. The three distinct clusters, Immunity High, Immunity Medium, and Immunity Low, showed different immune responses (Figure 1). The patient’s sample size of each subtype was 383 LUAD samples from Immunity High, 118 LUAD samples from Immunity Medium, and 34 samples from Immunity Low. The hierarchical clustering map was shown in Figure S1. Based on the estimation of stromal and immune cells in malignant tumor tissues using expression data (ESTIMATE) algorithm, the immune scores and stromal scores of Immunity High ranked the highest of the three groups, followed by that of Immunity Medium and Immunity Low (Figures 2A and 2B). Moreover, we compared the tumor purities of the three LUAD subtypes and obtained opposite trends: Immunity Low ranked the highest, and Immunity High ranked the lowest (Figure 2C). Using the CIBERSORT algorithm and combining it with the LM22 gene signature, the differences of immune infiltration among the different groups of LUAD patients of the 22 immune cell types were investigated. As shown in Figure 2D, the 22 tumor-immune cell proportions were significantly different. According to the boxplot, the Immunity High LUAD patients had notably higher proportions of CD8^+^ T cells (Figure 2E). These results showed that the heterogeneity of immune infiltration in LUAD may comprise targets for immunotherapy and may have significant clinical implications.

### Interaction between Immunogenomic Profiling-Based LUAD Subsets and the Expression of HLA (Human Leukocyte Antigen) and Immune Checkpoint Molecules

**Figure 3:**
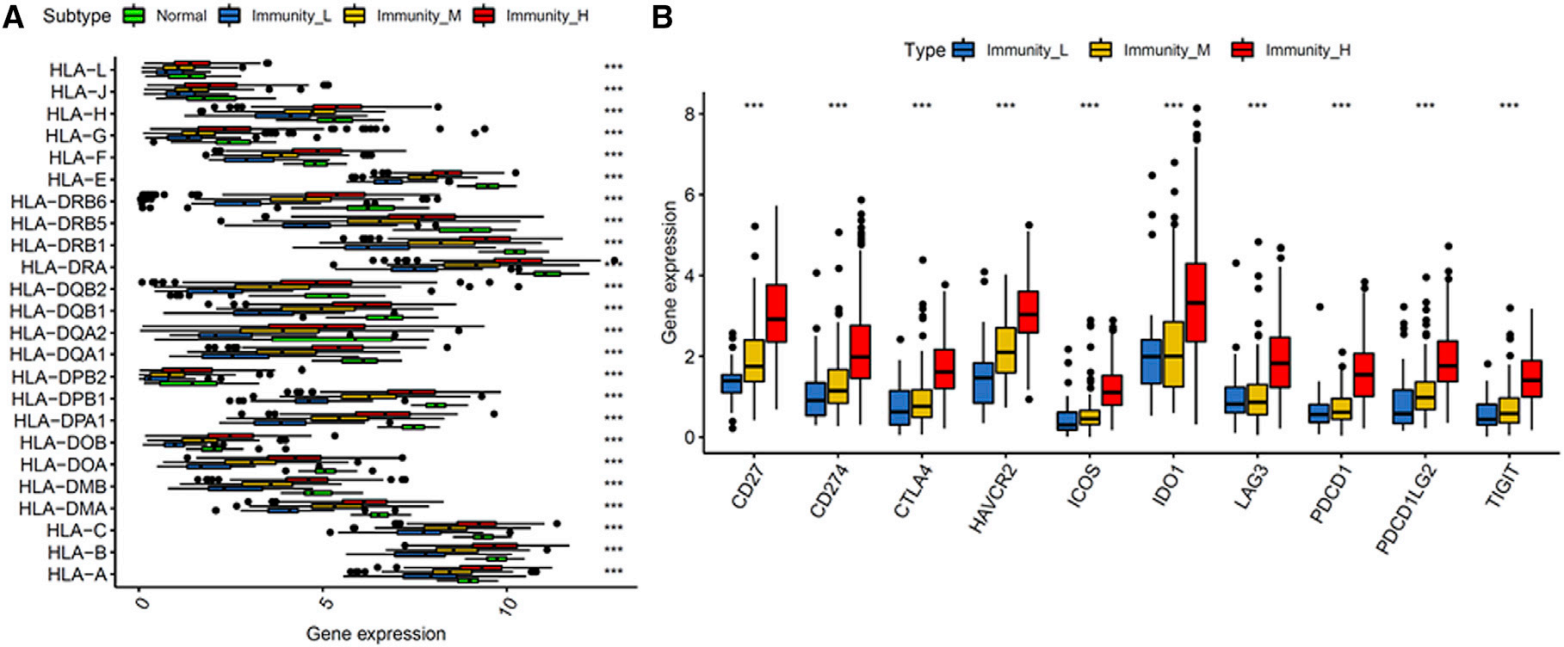
Interaction between Immunogenomic Profiling-Based LUAD Subsets and the Expression of HLA and Immune Checkpoint Molecules (A) The expression of HLA genes in LUAD subtypes. (B) The expression of immune checkpoint molecules in LUAD subtypes. HLA, human leukocyte antigen.

HLA and immune checkpoint molecules are essential for immune function and have diverse clinical implications in immunotherapy. Therefore, we investigated any potential correlation between the LUAD subtypes and the expression of HLA genes and immune checkpoint molecules. Interestingly, all HLA gene expression was enriched in Immunity High and exhibited the lowest expression levels in Immunity Low (Figure 3A). Then, we determined the expression of several key immunomodulators, including IDO1, PD-L1 (CD274), PD-L2 (PDCD1LG2), TIM-3 (HAVCR2), TIGIT, cytotoxic T-lymphocyte associated protein-4 (CTLA-4), PD-1 (PDCD1), LAG3, ICOS, and CD27. As shown in Figure 3B, Immunity High had greater expression of immune checkpoint molecules than the other two groups. These results revealed that the LUAD subtype Immunity High might be a more promising treatment to respond for immunotherapies.

### Association between the LUAD Subtypes and the Interferon-Gamma (IFNγ) Pathway

**Figure 4:**
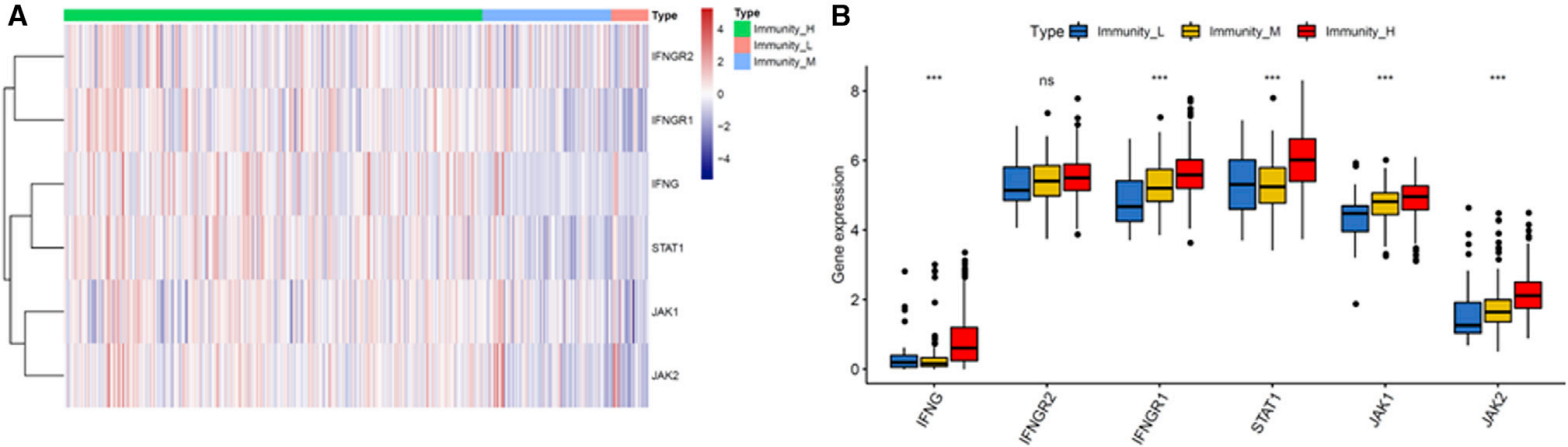
Association between LUAD Subtypes and the Th1/IFNγ Pathway (A) Heatmap demonstrating the relationship of LUAD subtypes with the markers of the Th1/IFNγ gene signature. (B) The expression of markers of the Th1/ IFNγ gene signature in LUAD subtypes. IFNγ, interferon-gamma.

In our study, we found Immunity High had elevated expression of CD8^+^ T cells, IDO1, and PD-1/PD-L1. An increasing amount of evidence reported that CD8^+^ T cells in the tumor microenvironment could produce IFNγ, leading to the upregulation of the PD-1/PD-L1 axis and IDO1. Therefore, we examined the markers of the T helper 1 (Th1)/IFNγ gene signature among the three immunity subtypes. Consistent with our hypothesis, a positive relationship between the immune response and IFNγ pathway-related genes could be seen, and Immunity High exhibited the highest IFNγ gene signature (Figures 4A and 4B).

### Association between the LUAD Subtypes and the Expression of *N*6-methyladenosine (m^6^A) Messenger RNA (mRNA) Methylation Regulators

**Figure 5:**
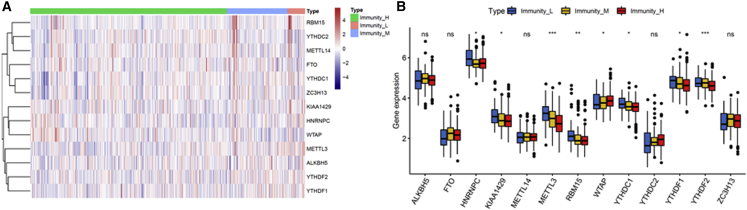
Association between LUAD Subtypes and the Expression of m^6^A mRNA Methylation Regulators (A) Heatmap demonstrating the relationship of LUAD subtypes with the expression of m^6^A mRNA methylation regulators. (B) The expression of markers of m^6^A mRNA methylation regulators in LUAD subtypes. m^6^A, *N*^6^-methyladenosine.

Emerging evidence revealed an important role of m^6^A mRNA methylation in decreasing the CD8^+^ T cell antitumor response and promoting anti-PD-1 resistance. Immunity High was significantly associated with decreased gene expression, such as METTL3, RBM15, YTHDC1, YTHDF1, and YTHDF2, which are involved in m6A mRNA methylation (Figures 5A and 5B). Our findings further demonstrate that patients in the Immunity High group might be better suited for immunotherapy in combination with emerging checkpoint inhibitors.

### Functional Annotation and Kyoto Encyclopedia of Genes and Genomes Analyses

**Figure 6:**
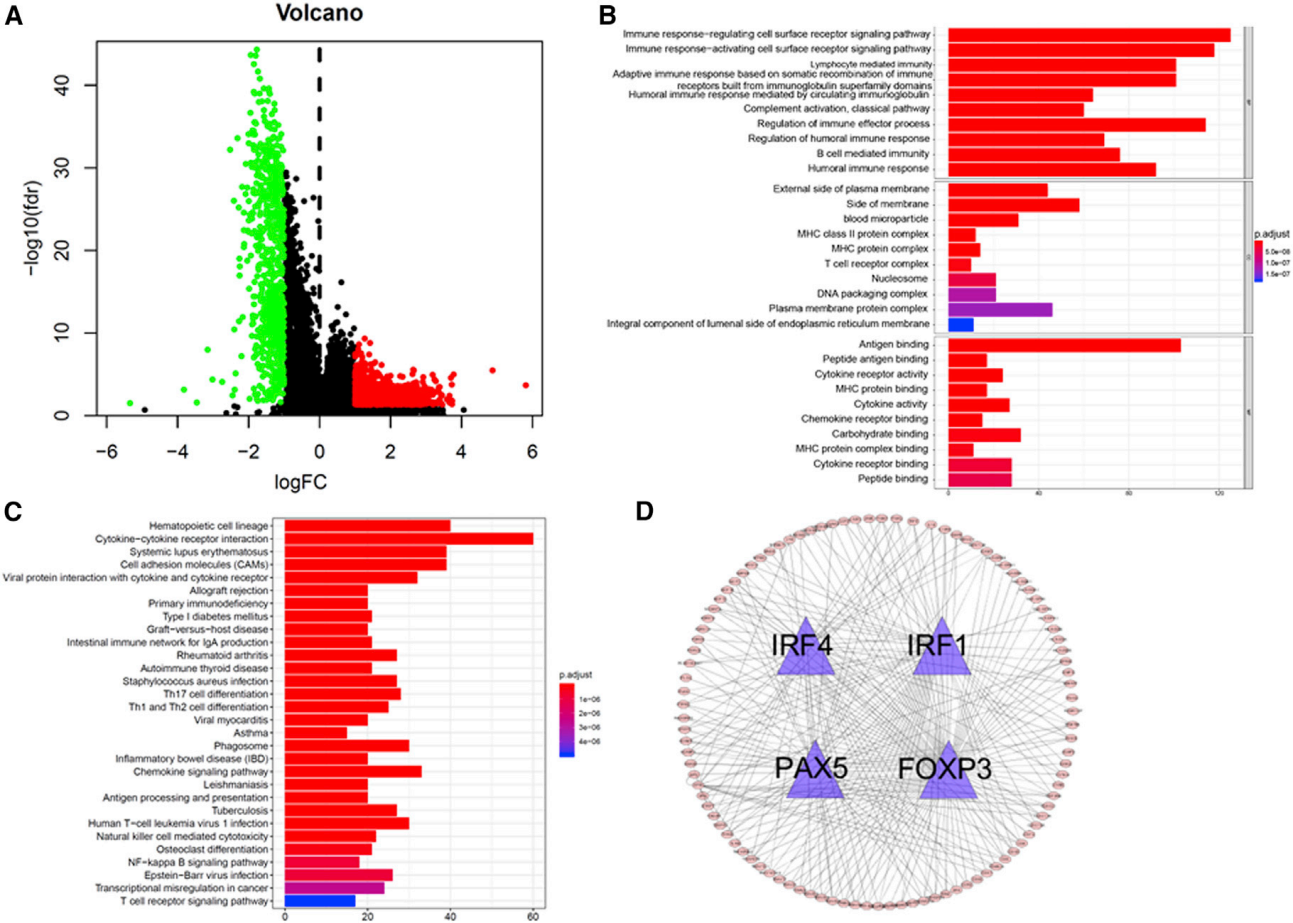
Functional Annotation and Kyoto Encyclopedia of Genes and Genomes Analyses (A) Volcano maps of differentially expressed genes. (B) GO enrichment analyses. (C) Kyoto Encyclopedia of Genes and Genomes (KEGG) pathways analyses. (D) TF genes and their regulated genes. TF, transcription factor.

Here, we found that the Immunity High subtype, compared with Immunity Medium or Immunity Low, was characterized by immune pathway, IFNγ pathway, HLA, and immune checkpoint molecule activation, and inactivation of m^6^A mRNA demethylation. Then, we compared the Immunity High group with the Immunity Medium and Immunity Low groups, and explored the differentially expressed genes using the limma package. A total of 1,710 differentially expressed genes were screened in The Cancer Genome Atlas (TCGA) dataset (Figure 6A).

In order to obtain further insight into the underlying biological characteristics of the differentially expressed genes, we conducted GO enrichment analyses based on the R package clusterProfiler. As a result, differentially expressed genes were clustered, and most were enriched in functions such as antigen binding, immune response-regulating cell surface receptor signaling pathway, immune response-activating cell surface receptor signaling pathway, lymphocyte-mediated immunity, adaptive immune response based on somatic recombination of immune receptors built from immunoglobulin superfamily domains, humoral immune response mediated by circulating immunoglobulin, regulation of immune effector process, regulation of humoral immune response, and B cell-mediated immunity (Figure 6B).

These biological functions indicated that the differentially expressed genes played an important role in immune-related biological processes in LUAD. Moreover, we identified various cancer-associated pathways that were enriched in cytokine-cytokine receptor interaction, cell adhesion molecules (CAMs), chemokine signaling pathway, nuclear factor κB (NF-κB) signaling pathway, transcriptional misregulation in cancer, and T cell receptor signaling pathway (Figure 6C). For the differentially expressed genes, we identified the four transcription factor (TF) genes, i.e., interferon regulatory factor 1 (IRF1), IRF4, PAX5, and FOXP3, all of which are involved in immune reactions (Figure 6D).

### Evaluating the Therapeutic Response of the LUAD Subtypes

**Figure 7:**
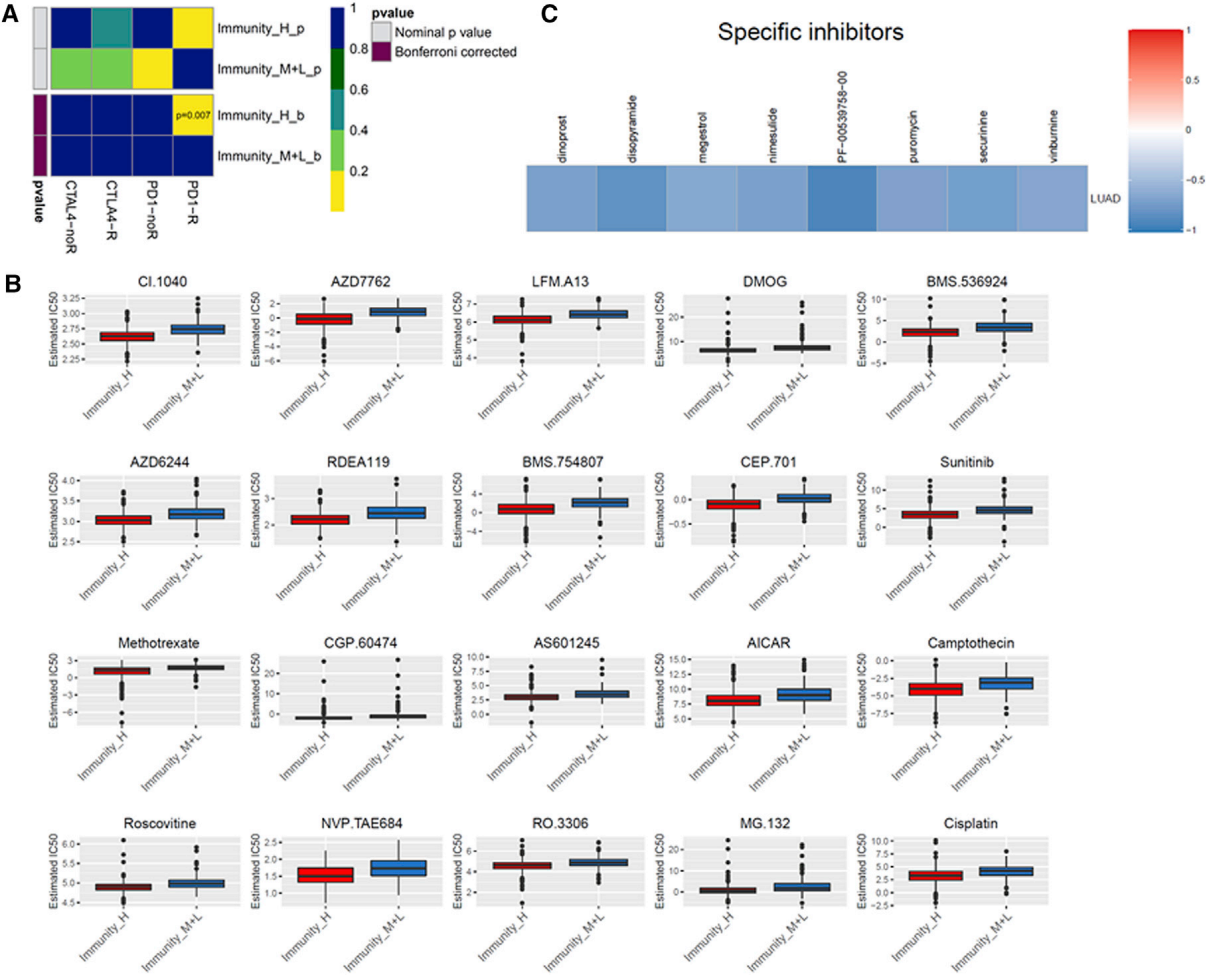
Evaluating the Therapeutic Response of the LUAD Subtypes (A) Differential immunotherapeutic response targeting CTLA-4 and PD-1 in LUAD subtypes. (B) Differential chemotherapeutic response based on IC_50_ available in the GDSC database in LUAD subtypes. (C) Heatmap showing enrichment score of each compound from the CMap. IC_50_, half-maximal inhibitory concentration.

Immune checkpoint blockade targeting CTLA-4 and PD-1 has emerged as a promising approach in treating a variety of malignancies. Thus, we used the Tumor Immune Dysfunction and Exclusion (TIDE) algorithm and subclass mapping to estimate the clinical response of the subtypes to immune checkpoint blockade (CTLA-4 and PD-1). Interestingly, we found that the Immunity High group was a more promising treatment to respond for anti-PD-1 therapy (Bonferroni corrected p = 0.004) (Figure 7A). To obtain a comprehensive analysis of the response to chemotherapy, we used the pRRophetic algorithm to estimate the chemotherapeutic response based on the half-maximal inhibitory concentration (IC_50_) available in the genomics of drug sensitivity in cancer (GDSC) database for each TCGA sample. We were delighted to find that 95 chemo drugs were screened out for significant differences in the estimated IC_50_ between the Immunity High group and the other two groups, and that the Immunity High group was more sensitive to all of these chemotherapies (Figure 7B; Table S1). Figure 7B displayed the top 20 chemo drugs. Next, we used a one-class logistic regression (OCLR) algorithm to calculate stemness indices across the LUAD subtypes. We found that the Immunity High subtype had a lower stemness index value than the other two subtypes (Figure S2).

Furthermore, a nomogram was built by including the TNM stage and the immune signature model (Figure S3A). As a result, the area under the curve (AUC) was the largest for immune signature score, indicating that the immune signature model was better than the clinical features in LUAD patients (Figure S3B). To explore the potential compounds/inhibitors that might target the immune signature, we used the Broad Institute’s Connectivity Map (CMap) based on differentially expressed genes. According to our analysis, we found some candidate compounds for LUAD patient treatment (Figure 7C).

## Discussion

Lung cancer, a deadly malignancy, ranks as the highest reason of global cancer mortality.^18^ Previous studies have identified LUAD subtypes according to genomic profiling;^19^, ^20^, ^21^, ^22^ however, very few studies have examined the classification of LUAD specifically on the basis of immune signatures. In order to better understand the immune biology components of LUAD, we classified LUAD into three subtypes: Immunity High, Immunity Medium, and Immunity Low. We demonstrated that the Immunity High subtype is associated with increased immune scores, stromal scores, HLA genes, immune checkpoint molecules, Th1/IFNγ gene signature, and the highest infiltration of CD8^+^ T cells, and decreased tumor purity and m6A RNA methylation. Functional and signaling pathway enrichment analysis further showed that differentially expressed genes between the Immunity High subtype and the other two subtypes mainly participated in the immune response and in some cancer-associated pathways. The Immunity High subtype exhibited more sensitivity to immunotherapy and chemotherapy. Our study, for the first time, stratified the LUAD patients based on immune signatures and provided novel insights into predicting the efficacy of immunotherapy and chemotherapy, as well as potential therapeutic targets for possible differentiation therapy.

Recently, immune checkpoint inhibitor therapy by targeting the PD-L1/PD-1 axis has provided promising approaches in the field of NSCLC therapy.^23^^,^^24^ CD8^+^ T cell-dependent killing of cancer cells requires the efficient cancer antigen presentation by HLA class I (HLA-I) molecules.^25^ CD8^+^ T cells could produce interferon Gamma (IFNG), then activate the expression of PD-1/PD-L1 as a consequence of antitumor immunity.^26^ m^6^A, the most prominent chemical mRNA modification, is responsible for mRNA post-transcriptional regulation in gene expression regulation.^27^ The role of m^6^A methylation in cancer has started to arouse wide concern in recent years. Increasing evidence indicates that genetic changes and dysregulated expression of m^6^A RNA are closely associated with tumor initiation, progression, and radio/chemo-resistance.^28^ m^6^A mRNA methylation was reported to decrease CD8^+^ T cell antitumor response and promote anti-PD-1 resistance.^29^ We hypothesized that the patients in different groups might have different immune responses. As expected in our study, we found that the Immunity High subtype generally had higher fractions of CD8^+^ T cells than the other two subtypes. Moreover, we found that the Immunity High subtype had elevated expression of HLA and immune checkpoint molecules, displayed a more prominent Th1/IFNγ gene signature, and had lower levels of m6A mRNA demethylation.

Although immune checkpoint inhibitors appear promising for lung cancer treatment, not all lung cancer patients respond to immune checkpoint inhibitors against PD-1 and CTLA-4, possibly because of their complexity and limitations in their tumor immunity.^30^^,^^31^ Thus, an improved classification of LUAD specifically based on immune signatures may reveal subsets of patients who may derive the most benefit from current therapies. Our results of functional and signaling pathway enrichment analysis mainly participated in the immune response and in some cancer-associated pathways. The TF genes interacting with each other and forming a subnetwork with immune and cancer-related genes that they regulate were involved in immune response. IRFs are a group of TFs that are related to the regulation of gene expression and the immune response.^32^ IRF1 has been found to have a central role in the immunologically active cancer phenotype.^33^ Its synthesis is induced in response to IFN-γ.^32^^,^^33^ Various genetic and functional studies have also pointed to IRF4 as a master regulator for autoimmunity. IRF4 can definitely affect CD8^+^ T cell differentiation because various factors related to the differentiation and function of CD8^+^ T cells, including basic leucine zipper ATF-like transcription factor (BATF), Blimp-1, T-bet, and retinoic acid-related orphan receptor gamma t (RORγt), are regulated by IRF4.^34^ In addition, IRF4 can affect T regulatory (Treg) cell development. Foxp3 modulated the expression of immune-associated molecules, and Foxp3 expression positively correlated with the Treg-like suppressive activity on T cells.^35^ Anti-PAX5-directed T cell therapy has potential clinical application in a range of adult and pediatric malignancies.^36^ Especially attractive is the prospect of generation of vectors for gene therapy encoding high-affinity T cell receptors directed against PAX5.^36^

We also used TIDE prediction and found that the Immunity High subtype was a more promising treatment to respond for anti-PD-1 therapy. Considering that chemotherapy is the common way to treat lung cancer, we used the pRRophetic algorithm to estimate the chemotherapeutic response based on IC_50_ available in the GDSC database for each TCGA sample. The results indicated that the Immunity High subtype was more sensitive to the chemotherapies than the other two subtypes. Then, we used CMap based on differentially expressed genes, and found candidate compounds for possible differentiation therapy of LUAD patients. Moreover, we found that the Immunity High subtype had lower stemness index values than the other two subtypes; higher values for stemness indices signal higher biological activity in cancer stem cells and greater tumor dedifferentiation.^37^ The above results implicate that the better prognosis with the Immunity High subtype may be because of a higher immunoreactive environment and because it inhibits tumor growth, progression, invasion, and metastasis. In addition, the Immunity High subtype may benefit more from immunotherapy and chemotherapy determined by these differences.

Our research provides new insights into the LUAD immune microenvironment. However, our research was limited because it was retrospective, and our results should thus be further confirmed by prospective studies. Additionally, the TCGA data enrolled for analysis were mostly collected from patients in developed countries but lacked data from developing countries.

Overall, for the first time, our study may provide a better assessment of the immune signature-based classification of LUAD. Our findings also infer potential treatments for the development of immunotherapeutic and chemotherapeutic strategies, and may guide the development of novel drug strategies.

## Materials and Methods

### Data Source

Gene expression data and the corresponding clinical features for LUAD patients were accessed from TCGA website. This study meets TCGA’s publication guidelines. All of the LUAD gene expression and clinical data were downloaded as determined by the Data Coordinating Center (DCC).

### Hierarchical Clustering of LUAD Patients

To quantify the proportions of immune signatures in the LUAD samples based on the ssGSEA score, we used the 29 immune signatures, including cell types, functions, and pathways.^9^

### Evaluation of Immune Microenvironment

Immune score and stromal score were evaluated by applying the ESTIMATE algorithm to the gene expression data from TCGA.^38^^,^^39^ Tumor purity was obtained based on the ESTIMATE score using a fitted formula as previously described.^39^

### Screening of Differentially Expressed Genes

The raw counts of TCGA gene expression were normalized and determined by a weighted trimmed mean of log ratios-based method.^40^ In order to obtain differentially expressed genes, R package “limma” using the standard comparison mode was performed.^41^ The threshold was determined as |log2 fold change (log2FC)| ≥ 1 and false discovery rate (FDR) < 0.05.

### Functional and Pathway Enrichment Analysis

Gene Ontology (GO) and the Kyoto Encyclopedia of Genes and Genomes (KEGG) pathway analysis using the clusterProfiler R package were performed on differentially expressed genes.^42^ The thresholds for analyses were determined by a p value <0.05, indicating significantly enriched functional annotations.

### Estimation of Tumor-Infiltrating Immune Cells

We uploaded the normalized gene expression data with standard annotation files to the CIBERSORT web portal, and the algorithm was determined by 1,000 permutations and by the LM22 gene signature as described in previous literature.^43^^,^^44^ The R “Genefilter” package was applied to screen each sample, and the threshold was determined as p value <0.05.

### Immunotherapeutic and Chemotherapeutic Response Prediction

The PD-1/PD-L1 and CTLA-4 pathways in cancer are implicated in tumors escaping immune destruction; thus, immune checkpoint inhibitors targeting PD-1 and CTLA-4 enhance antitumor immunity.^45^ Here, in order to predict the clinical response to immune checkpoint inhibitors, we ran the TIDE algorithm and subclass mapping as described previously.^46^ Considering that chemotherapy is a common clinical practice to treat NSCLC, we applied the R package pRRophetic to estimate the chemotherapeutic response determined by the IC_50_ for each LUAD patient on the GDSC website.^47^^,^^48^

### Calculation of Stemness Index

Stemness indices were calculated using an innovative OCLR machine-learning algorithm as previously described.^14^^,^^38^ Then, we calculated Spearman correlations between the stemness index model and the lung cancer sample’s expression profile from TCGA. The stemness indices were subsequently mapped to the [0,1] range via utilizing a linear transformation that subtracted the minimum and divided by the maximum.

### Compounds Therapeutic Response Prediction

To identify which target compounds might be useful, we used the CMap in predicting which compounds based on the top 1,000 differentially expressed genes.^14^

### Statistical Analysis

All statistical analyses were performed using R version 3.6.1, and the data from different groups were compared by Mann-Whitney-Wilcoxon test. Pearson’s chi-square test was performed to measure the level of significance for association among variables. All reported p values were two-tailed, and p < 0.05 was considered statistically significant.

## Author Contributions

F.X. and L.L. designed the study, analyzed data, and wrote the manuscript. J.-x.C., X.-b.Y., X.-b.H., and Z.-x.L. analyzed data and contributed in writing the manuscript. Y.-s.C. supervised the research, analyzed data, and wrote the manuscript.

## Conflicts of Interest

The authors declare no competing interests.

## Supplemental Information

Document S1. Figures S1–S3 and Table S1

Document S2. Article plus Supplemental Information
