# Supplementary material for: Analysis of Lung Adenocarcinoma Subtypes Based on Immune Signatures Identifies Clinical Implications for Cancer Therapy
Source: Mol Ther Oncolytics. 2020 Apr 7;17:241–9. doi: 10.1016/j.omto.2020.03.021 (PMC7183104; doi:10.1016/j.omto.2020.03.021)
Supplement: Document S1. Figures S1–S3 and Table S1 [file mmc1.pdf]

OMTO, Volume *17*

Supplemental Information

Analysis of Lung Adenocarcinoma Subtypes Based on Immune Signatures Identifies Clinical Implications for Cancer Therapy

Feng Xu, Jie-xin Chen, Xiong-bin Yang, Xin-bin Hong, Zi-xiong Li, Ling Lin, and Yong-song Chen

Supplemental Figure 1 Hierarchical clustering of LUAD patients.

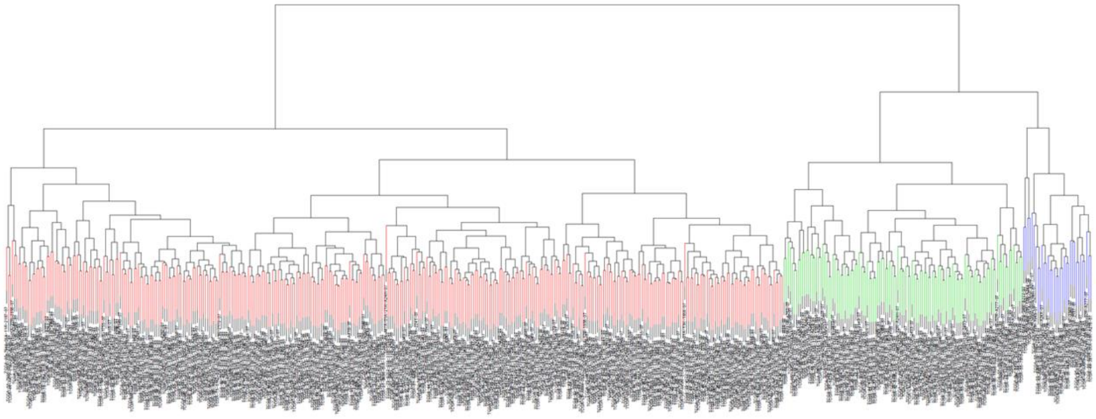

Supplemental Figure 2 Stemness indices in LUAD subtypes.

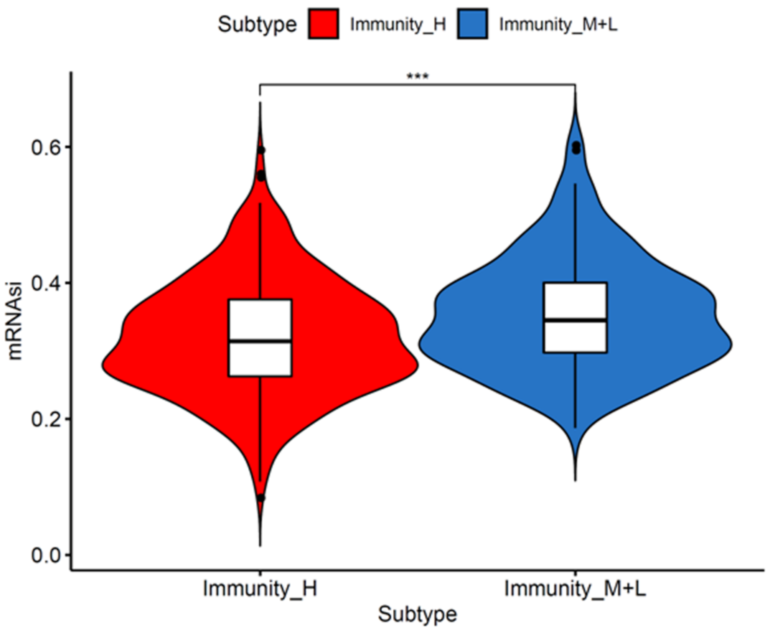

Supplemental Figure 3 Relationship between the immune signature model and the clinical information. (A) Nomogram for predicting the probability of 1-, 3-, and 5-year overall survival for LUAD patients.; (B) Time-dependent ROC curve analyses of the immune signature model, gender, TNM stage, T stage, N stage, M stage.

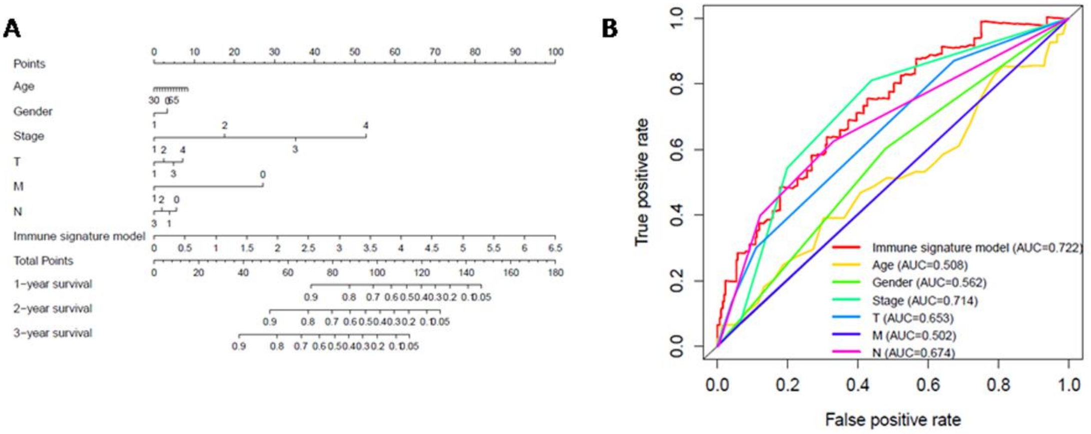

Supplemental table 1 chemotherapeutic response

GDSC name	p	Synonyms	Targets	Target way
CI.1040	2.04E-23	CI 1040, PD-18435, PD-184352, 212631-79-3	MEK1, MEK2	ERK MAPK signaling
AZD7762	4.84E-23	AZD-7762, AZD 7762	CHEK1, CHEK2	Cell cycle
LFM.A13	3.93E-22	DDE-28	BTK	Other, kinases
DMOG	4.53E-20	Dimethyloxalylglcine	HIF-PH	Metabolism
BMS.536924	1.22E-18	BMS 536924	IGF1R, IR	IGF1R signaling
AZD6244	1.98E-18	AZD6244, AZD-6244, ARRY-886，Selumetinib	MEK1, MEK2	ERK MAPK signaling
RDEA119	2.01E-18	RDEA119, BAY-86-9766, BAY 869766	MEK1, MEK2	ERK MAPK signaling
BMS.754807	2.17E-17	BMS754807, BMS 754807	IGF1R, IR	RTK signaling
CEP.701	2.55E-17	CEP-701, SP-924, SPM-924, A-154475, KT-555，Lestaurtinib	FLT3, JAK2, NTRK1, NTRK2, NTRK3	Other, kinases
Sunitinib	3.45E-17	Sutent, Sunitinib Malate, SU-11248	PDGFR, KIT, VEGFR, FLT3, RET, CSF1R	RTK signaling
Methotrexate	4.41E-15	Abitrexate, Amethopterin, Rheumatrex, Trexall, Folex	Antimetabolite	DNA replication
CGP.60474	6.33E-15	KIN001-019, CGP60474, CGP 60474	CDK1,CDK2,CDK5,CDK7,CDK9, PKC	Cell cycle
AS601245	2.89E-13	/	JNK1, JNK2, JNK2	JNK and p38 signaling
AICAR	3.81E-13	AICAR, N1-(b-D-Ribofuranosyl)-5-aminoimidazole-4-carboxamide	AMPK agonist	Metabolism
Camptothecin	8.97E-13	Camptothecine, (+)-Camptothecin	TOP1	DNA replication
Roscovitine	1.69E-12	Roscovitine, CYC-202, AL-39256	CDK2, CDK7, CDK9	Cell cycle
NVP.TAE684	2.05E-12	NVP-TAE 684, TAE684, TAE-684	ALK	RTK signaling
RO.3306	7.05E-12	/	CDK1	Cell cycle
Cisplatin	1.27E-11	cis-Diammineplatinum(II) dichloride, Platinol, CIS-DDP	DNA crosslinker	DNA replication
MG.132	1.27E-11	LLL cpd, MG 132, MG132	Proteasome, CAPN1	Protein stability and degradation
