# Supplementary material for: Analysis of Lung Adenocarcinoma Subtypes Based on Immune Signatures Identifies Clinical Implications for Cancer Therapy
Source: Mol Ther Oncolytics. 2020 Apr 7;17:241–9. doi: 10.1016/j.omto.2020.03.021 (PMC7183104; doi:10.1016/j.omto.2020.03.021)
Supplement: Document S2. Article plus Supplemental Information [file mmc2.pdf]

Molecular Therapy
Oncolytics
Original Article

Analysis of Lung Adenocarcinoma Subtypes Based on Immune Signatures Identifies Clinical Implications for Cancer Therapy

Feng Xu,[1] Jie-xin Chen,[2] Xiong-bin Yang,[3] Xin-bin Hong,[2] Zi-xiong Li,[3] Ling Lin,[3] and Yong-song Chen[2]

[1]Department of Respiratory Medicine, The First Affiliated Hospital of Shantou University Medical College, No. 57 Changping Road, Shantou, Guangdong 515041, P.R. China; [2]Department of Endocrinology, The First Affiliated Hospital of Shantou University Medical College, No. 57 Changping Road, Shantou, Guangdong 515041, P.R. China; [3]Department of Rheumatology, The First Affiliated Hospital of Shantou University Medical College, No. 57 Changping Road, Shantou, Guangdong 515041, P.R. China

Lung cancer is the most common cause of cancer deaths worldwide, and lung adenocarcinoma (LUAD) is the most common histological subtype. However, the prognostic and predictive outcomes differ because of this cancer type heterogeneity. LUAD subtypes were identified on the basis of the immunogenomic profiling of 29 immune signatures. We named three LUAD subtypes: Immunity High, Immunity Medium, and Immunity Low. The Immunity High subtype was characterized by immune activation, e.g., increased immune scores, elevated stromal scores and the highest infiltration of $CD8^+$ T cells, and decreased tumor purities. Activated expressions of human leukocyte antigen (HLA) genes, immune checkpoint molecules, and T helper 1 (Th1)/interferon-gamma (IFNγ) gene signature were also observed in the Immunity High subtype. N^6-methyladenosine (m^6A) RNA methylation, associated with cancer initiation and progression, was reduced in the Immunity High subtype. Functional and signaling pathway enrichment analysis further showed that differentially expressed genes between the Immunity High subtype and the other subtypes mainly participated in immune response and some cancer-associated pathways. In addition, the Immunity High subtype exhibited more sensitivity to immunotherapy and chemotherapy. Finally, candidate compounds that aimed at LUAD subtype differentiation were identified. Comprehensively characterizing the LUAD subtypes based on immune signatures may help to provide potential strategies for LUAD treatment.

INTRODUCTION

Lung cancer is the leading cause of cancer-associated mortality worldwide.[1,2] Although great progress has been made toward the prevention, diagnosis, and treatment of cancer via specific cellular targets, the clinical outcome is still unsatisfactory. An increasing body of evidence reports that malignant phenotypes are influenced by a tumor-related microenvironment.[3,4] Lung cancer, an immune-sensitive malignancy, is infiltrated by different immune cell types.[5] Recently, cancer immunotherapy has become involved in treating all forms of cancer and has changed the landscape of cancer care. For example, inhibition of the programmed cell death 1 (PCDC1/PD-1)/CD274 molecule (CD274/PD-L1) immune checkpoint using antibodies against PD-1 rescues effector T cell function, which permits T cells to maintain their tumor cell-killing function.[6] Moreover, in patients with high expression of PD-L1, antibodies against PD-1 are effective in treating a variety of cancers and improving overall survival.[7,8] However, currently, cancer immunotherapy displays beneficial effects in less than 20% of patients.[9] This may suggest that not all cancer patients could respond to immunotherapy. Lung adenocarcinoma (LUAD) is one of the major types of lung cancer, and a recent study identified an immunogenic tumor microenvironment state in non-small cell lung cancer (NSCLC) that was mainly enriched for the LUAD subtype.[10] Also, many studies identified distinct subtypes of LUAD featured by different immune-infiltrating signatures and molecular mechanisms.[11,12] The 5-year overall survival rate of LUAD remains at a low level of 15.9%.[13] Therefore, it is essential to identify the LUAD subtypes based on immune signature.

In the present study, we classified LUAD into three distinct subtypes based on immunogenomic profiling: Immunity Low, Immunity Medium, and Immunity High. Furthermore, our analyses apply a new approach of identifying the optimal selection of LUAD patients responsive to immunotherapy and chemotherapy, and may provide a predictive factor for clinical application in LUAD patient treatment. Finally, recent pharmacology research has revealed the necessity to design compounds that act on multiple genes or molecular pathways.[14–17] In our study, we identified compounds targeting the differentiation of LUAD phenotypes, which may provide therapeutic targets for further analysis.

Received 23 February 2020; accepted 26 March 2020;
https://doi.org/10.1016/j.omto.2020.03.021.

Correspondence: Yong-song Chen, Department of Endocrinology, The First Affiliated Hospital of Shantou University Medical College, No. 57 Changping Road, Shantou, Guangdong 515041, P.R. China.
E-mail: yongsongchen@126.com
Correspondence: Ling Lin, Department of Rheumatology, The First Affiliated Hospital of Shantou University Medical College, No. 57 Changping Road, Shantou, Guangdong 515041, P.R. China.
E-mail: llinc@163.net

This is an open access article under the CC BY license (http://creativecommons.org/licenses/by/4.0/).

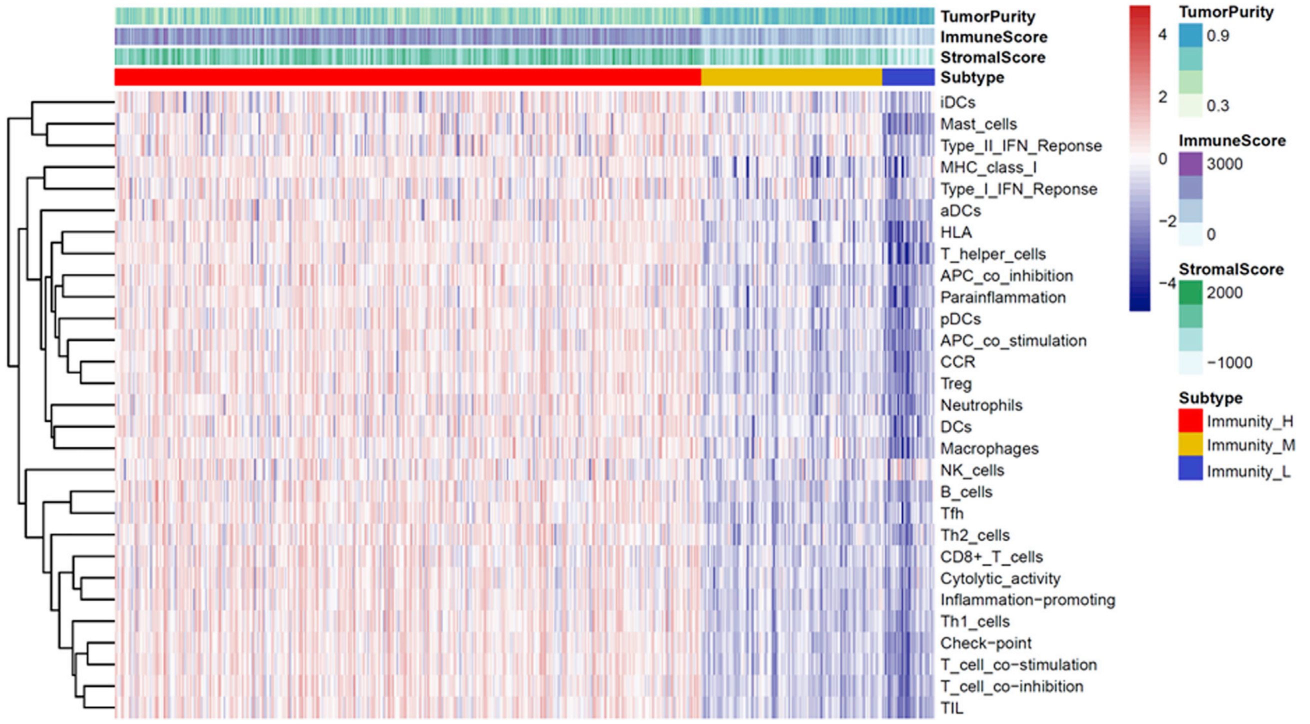

Figure 1. Hierarchical Clustering Yields Three Subtypes in TCGA Dataset
Immunity_H, Immunity High; Immunity_L, Immunity Low; Immunity_M, Immunity Medium; LUAD, lung adenocarcinoma.

RESULTS

Identification of LUAD Subtypes Based on Immunogenomic Profiling

To characterize the immune subtypes and immune response to cancer in LUAD patients, we analyzed the single-sample gene set enrichment analysis (ssGSEA) score using 29 immune-associated gene sets across the landscape of LUAD samples. Subsequent hierarchical cluster analysis identified characteristic immunoncological signatures, which were then used to cluster LUAD tumor types into immune subtypes. The three distinct clusters, Immunity High, Immunity Medium, and Immunity Low, showed different immune responses (Figure 1). The patient's sample size of each subtype was 383 LUAD samples from Immunity High, 118 LUAD samples from Immunity Medium, and 34 samples from Immunity Low. The hierarchical clustering map was shown in Figure S1. Based on the estimation of stromal and immune cells in malignant tumor tissues using expression data (ESTIMATE) algorithm, the immune scores and stromal scores of Immunity High ranked the highest of the three groups, followed by that of Immunity Medium and Immunity Low (Figures 2A and 2B). Moreover, we compared the tumor purities of the three LUAD subtypes and obtained opposite trends: Immunity Low ranked the highest, and Immunity High ranked the lowest (Figure 2C). Using the CIBERSORT algorithm and combining it with the LM22 gene signature, the differences of immune infiltration among the different groups of LUAD patients of the 22 immune cell types were investigated. As shown in Figure 2D, the 22 tumor-immune cell proportions were significantly different. According to the boxplot, the Immunity High LUAD patients had notably higher proportions of $CD8^+$ T cells (Figure 2E). These results showed that the heterogeneity of immune infiltration in LUAD may comprise targets for immunotherapy and may have significant clinical implications.

Interaction between Immunogenomic Profiling-Based LUAD Subsets and the Expression of HLA (Human Leukocyte Antigen) and Immune Checkpoint Molecules

HLA and immune checkpoint molecules are essential for immune function and have diverse clinical implications in immunotherapy. Therefore, we investigated any potential correlation between the LUAD subtypes and the expression of HLA genes and immune checkpoint molecules. Interestingly, all HLA gene expression was enriched in Immunity High and exhibited the lowest expression levels in Immunity Low (Figure 3A). Then, we determined the expression of several key immunomodulators, including IDO1, PD-L1 (CD274), PD-L2 (PDCD1LG2), TIM-3 (HAVCR2), TIGIT, cytotoxic T-lymphocyte associated protein-4 (CTLA-4), PD-1 (PDCD1), LAG3, ICOS, and CD27. As shown in Figure 3B, Immunity High had greater expression of immune checkpoint molecules than the other two groups. These results revealed that the LUAD subtype Immunity High might be a more promising treatment to respond for immunotherapies.

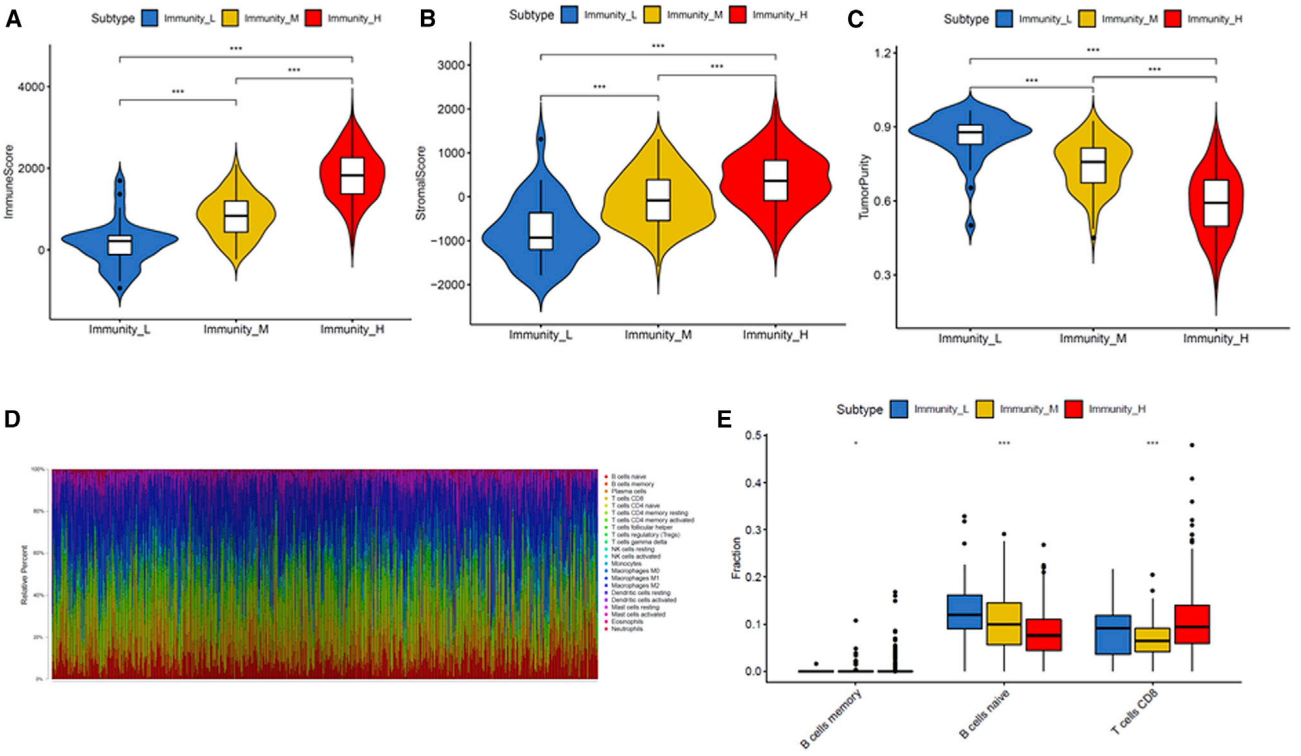

Figure 2. The Landscape of Immune Infiltration in LUAD Subtypes

(A) Immune scores in LUAD subtypes. (B) Stromal scores in LUAD subtypes. (C) Tumor purities in LUAD subtypes. (D) Relative proportion of immune infiltration in LUAD subtypes. (E) The difference of immune cell infiltration abundance in LUAD subtypes.

Association between the LUAD Subtypes and the Interferon-Gamma (IFNγ) Pathway

In our study, we found Immunity High had elevated expression of $CD8^+$ T cells, IDO1, and PD-1/PD-L1. An increasing amount of evidence reported that $CD8^+$ T cells in the tumor microenvironment could produce IFNγ, leading to the upregulation of the PD-1/PD-L1 axis and IDO1. Therefore, we examined the markers of the T helper 1 (Th1)/IFNγ gene signature among the three immunity subtypes. Consistent with our hypothesis, a positive relationship between the immune response and IFNγ pathway-related genes could be seen, and Immunity High exhibited the highest IFNγ gene signature (Figures 4A and 4B).

Association between the LUAD Subtypes and the Expression of *N*6-methyladenosine (m^6A) Messenger RNA (mRNA) Methylation Regulators

Emerging evidence revealed an important role of m^6A mRNA methylation in decreasing the $CD8^+$ T cell antitumor response and promoting anti-PD-1 resistance. Immunity High was significantly associated with decreased gene expression, such as METTL3, RBM15, YTHDC1, YTHDF1, and YTHDF2, which are involved in m6A mRNA methylation (Figures 5A and 5B). Our findings further demonstrate that patients in the Immunity High group might be better suited for immunotherapy in combination with emerging checkpoint inhibitors.

Functional Annotation and Kyoto Encyclopedia of Genes and Genomes Analyses

Here, we found that the Immunity High subtype, compared with Immunity Medium or Immunity Low, was characterized by immune pathway, IFNγ pathway, HLA, and immune checkpoint molecule activation, and inactivation of m^6A mRNA demethylation. Then, we compared the Immunity High group with the Immunity Medium and Immunity Low groups, and explored the differentially expressed genes using the limma package. A total of 1,710 differentially expressed genes were screened in The Cancer Genome Atlas (TCGA) dataset (Figure 6A).

In order to obtain further insight into the underlying biological characteristics of the differentially expressed genes, we conducted GO enrichment analyses based on the R package clusterProfiler. As a result, differentially expressed genes were clustered, and most were enriched in functions such as antigen binding, immune response-regulating cell surface receptor signaling pathway, immune response-activating cell surface receptor signaling pathway, lymphocyte-mediated immunity, adaptive immune response based on somatic recombination of immune receptors built from immunoglobulin superfamily domains, humoral immune response mediated by circulating immunoglobulin, regulation of immune effector process, regulation of humoral immune response, and B cell-mediated immunity (Figure 6B).

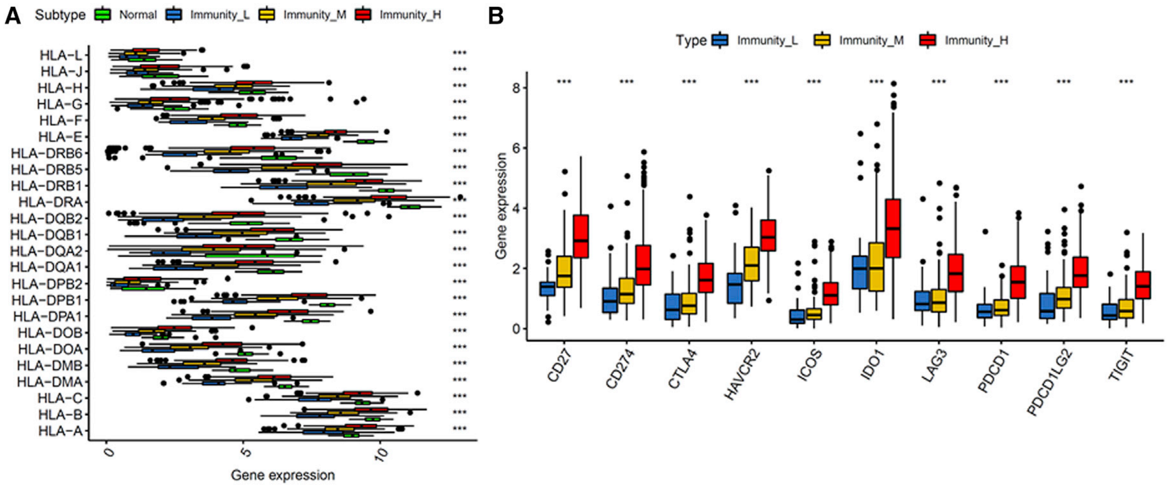

Figure 3. Interaction between Immunogenomic Profiling-Based LUAD Subsets and the Expression of HLA and Immune Checkpoint Molecules

(A) The expression of HLA genes in LUAD subtypes. (B) The expression of immune checkpoint molecules in LUAD subtypes. HLA, human leukocyte antigen.

These biological functions indicated that the differentially expressed genes played an important role in immune-related biological processes in LUAD. Moreover, we identified various cancer-associated pathways that were enriched in cytokine-cytokine receptor interaction, cell adhesion molecules (CAMs), chemokine signaling pathway, nuclear factor κB (NF-κB) signaling pathway, transcriptional misregulation in cancer, and T cell receptor signaling pathway (Figure 6C). For the differentially expressed genes, we identified the four transcription factor (TF) genes, i.e., interferon regulatory factor 1 (IRF1), IRF4, PAX5, and FOXP3, all of which are involved in immune reactions (Figure 6D).

Evaluating the Therapeutic Response of the LUAD Subtypes

Immune checkpoint blockade targeting CTLA-4 and PD-1 has emerged as a promising approach in treating a variety of malignancies. Thus, we used the Tumor Immune Dysfunction and Exclusion (TIDE) algorithm and subclass mapping to estimate the clinical response of the subtypes to immune checkpoint blockade (CTLA-4 and PD-1). Interestingly, we found that the Immunity High group was a more promising treatment to respond for anti-PD-1 therapy (Bonferroni corrected $p = 0.004$) (Figure 7A). To obtain a comprehensive analysis of the response to chemotherapy, we used the pRRophetic algorithm to estimate the chemotherapeutic response based on the half-maximal inhibitory concentration (IC_{50}) available in the genomics of drug sensitivity in cancer (GDSC) database for each TCGA sample. We were delighted to find that 95 chemo drugs were screened out for significant differences in the estimated IC_{50} between the Immunity High group and the other two groups, and that the Immunity High group was more sensitive to all of these chemotherapies (Figure 7B; Table S1). Figure 7B displayed the top 20 chemo drugs. Next, we used a one-class logistic regression (OCLR) algorithm to calculate stemness indices across the LUAD subtypes. We found that the Immunity High subtype had a lower stemness index value than the other two subtypes (Figure S2).

Furthermore, a nomogram was built by including the TNM stage and the immune signature model (Figure S3A). As a result, the area under the curve (AUC) was the largest for immune signature score, indicating that the immune signature model was better than the clinical features in LUAD patients (Figure S3B). To explore the potential compounds/inhibitors that might target the immune signature, we used the Broad Institute's Connectivity Map (CMap) based on differentially expressed genes. According to our analysis, we found some candidate compounds for LUAD patient treatment (Figure 7C).

DISCUSSION

Lung cancer, a deadly malignancy, ranks as the highest reason of global cancer mortality.[18] Previous studies have identified LUAD subtypes according to genomic profiling;[19–22] however, very few studies have examined the classification of LUAD specifically on the basis of immune signatures. In order to better understand the immune biology components of LUAD, we classified LUAD into three subtypes: Immunity High, Immunity Medium, and Immunity Low. We demonstrated that the Immunity High subtype is associated with increased immune scores, stromal scores, HLA genes, immune checkpoint molecules, Th1/IFNγ gene signature, and the highest infiltration of $CD8^+$ T cells, and decreased tumor purity and m6A RNA methylation. Functional and signaling pathway enrichment analysis further showed that differentially expressed genes between the Immunity High subtype and the other two subtypes mainly participated in the immune response and in some cancer-associated pathways. The Immunity High subtype exhibited more sensitivity to immunotherapy and chemotherapy. Our study, for the first time, stratified the LUAD patients based on immune signatures and provided novel insights into predicting the efficacy of immunotherapy and chemotherapy, as well as potential therapeutic targets for possible differentiation therapy.

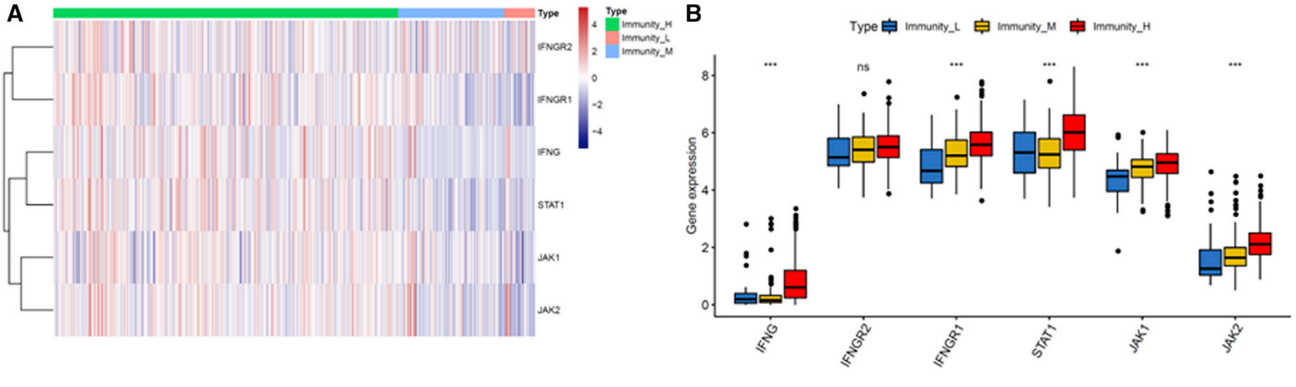

Figure 4. Association between LUAD Subtypes and the Th1/IFNγ Pathway

(A) Heatmap demonstrating the relationship of LUAD subtypes with the markers of the Th1/IFNγ gene signature. (B) The expression of markers of the Th1/ IFNγ gene signature in LUAD subtypes. IFNγ, interferon-gamma.

Recently, immune checkpoint inhibitor therapy by targeting the PD-L1/PD-1 axis has provided promising approaches in the field of NSCLC therapy.[23,24] CD8$^+$ T cell-dependent killing of cancer cells requires the efficient cancer antigen presentation by HLA class I (HLA-I) molecules.[25] CD8$^+$ T cells could produce interferon Gamma (IFNG), then activate the expression of PD-1/PD-L1 as a consequence of antitumor immunity.[26] m^{6}A, the most prominent chemical mRNA modification, is responsible for mRNA post-transcriptional regulation in gene expression regulation.[27] The role of m^{6}A methylation in cancer has started to arouse wide concern in recent years. Increasing evidence indicates that genetic changes and dysregulated expression of m^{6}A RNA are closely associated with tumor initiation, progression, and radio/chemo-resistance.[28] m^{6}A mRNA methylation was reported to decrease CD8$^+$ T cell antitumor response and promote anti-PD-1 resistance.[29] We hypothesized that the patients in different groups might have different immune responses. As expected in our study, we found that the Immunity High subtype generally had higher fractions of CD8$^+$ T cells than the other two subtypes. Moreover, we found that the Immunity High subtype had elevated expression of HLA and immune checkpoint molecules, displayed a more prominent Th1/IFNγ gene signature, and had lower levels of m6A mRNA demethylation.

Although immune checkpoint inhibitors appear promising for lung cancer treatment, not all lung cancer patients respond to immune checkpoint inhibitors against PD-1 and CTLA-4, possibly because of their complexity and limitations in their tumor immunity.[30,31] Thus, an improved classification of LUAD specifically based on immune signatures may reveal subsets of patients who may derive the most benefit from current therapies. Our results of functional and signaling pathway enrichment analysis mainly participated in the immune response and in some cancer-associated pathways. The TF genes interacting with each other and forming a subnetwork with immune and cancer-related genes that they regulate were involved in immune response. IRFs are a group of TFs that are related to the regulation of gene expression and the immune response.[32] IRF1 has been found to have a central role in the immunologically active cancer phenotype.[33] Its synthesis is induced in response to IFN-γ.[32,33] Various genetic and functional studies have also pointed to IRF4 as a master regulator for autoimmunity.

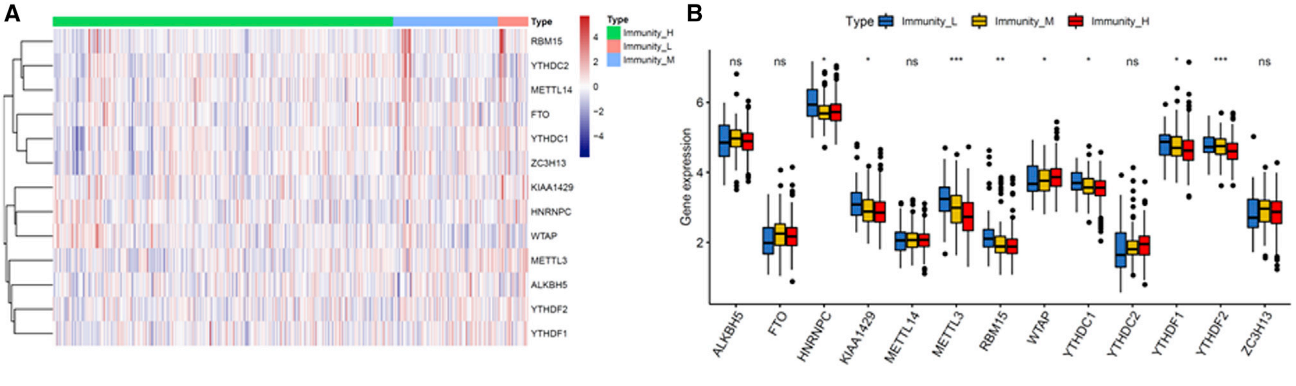

Figure 5. Association between LUAD Subtypes and the Expression of m^{6}A mRNA Methylation Regulators

(A) Heatmap demonstrating the relationship of LUAD subtypes with the expression of m^{6}A mRNA methylation regulators. (B) The expression of markers of m^{6}A mRNA methylation regulators in LUAD subtypes. m^{6}A, N^6-methyladenosine.

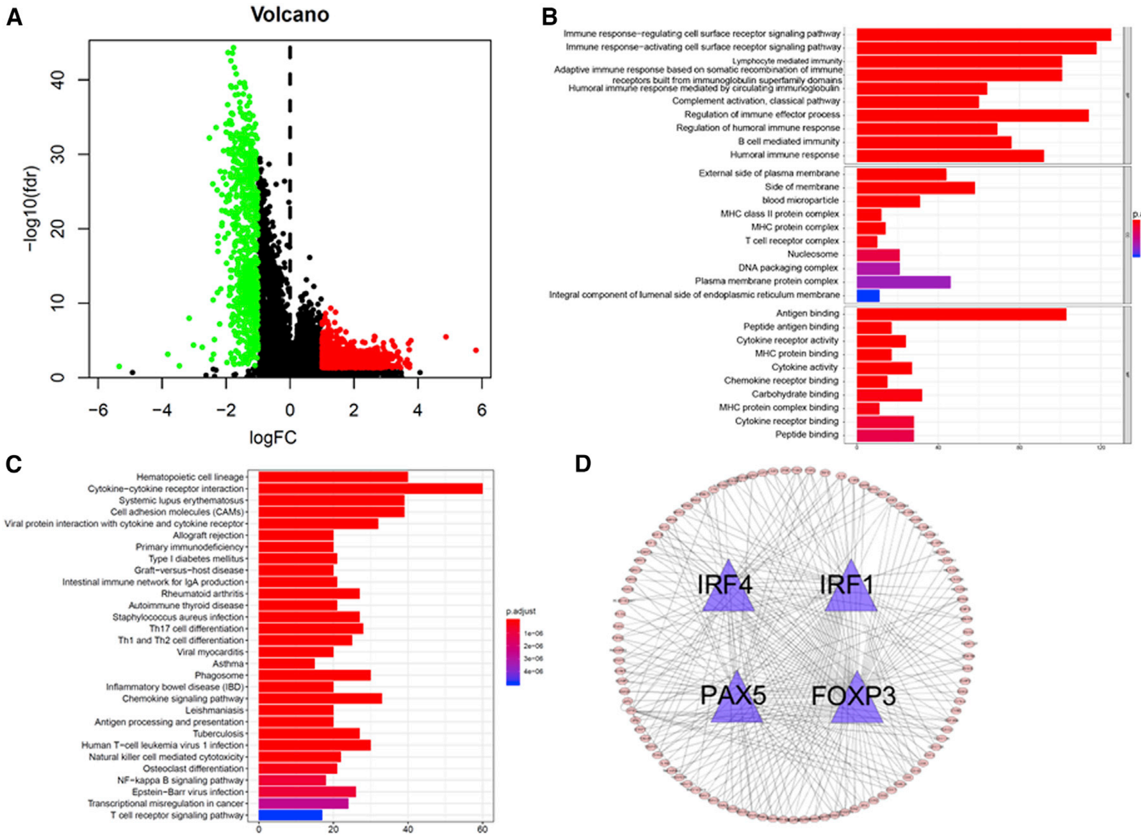

Figure 6. Functional Annotation and Kyoto Encyclopedia of Genes and Genomes Analyses

(A) Volcano maps of differentially expressed genes. (B) GO enrichment analyses. (C) Kyoto Encyclopedia of Genes and Genomes (KEGG) pathways analyses. (D) TF genes and their regulated genes. TF, transcription factor.

IRF4 can definitely affect $CD8^+$ T cell differentiation because various factors related to the differentiation and function of $CD8^+$ T cells, including basic leucine zipper ATF-like transcription factor (BATF), Blimp-1, T-bet, and retinoic acid-related orphan receptor gamma t (RORγt), are regulated by IRF4.[34] In addition, IRF4 can affect T regulatory (Treg) cell development. Foxp3 modulated the expression of immune-associated molecules, and Foxp3 expression positively correlated with the Treg-like suppressive activity on T cells.[35] Anti-PAX5-directed T cell therapy has potential clinical application in a range of adult and pediatric malignancies.[36] Especially attractive is the prospect of generation of vectors for gene therapy encoding high-affinity T cell receptors directed against PAX5.[36]

We also used TIDE prediction and found that the Immunity High subtype was a more promising treatment to respond for anti-PD-1 therapy. Considering that chemotherapy is the common way to treat lung cancer, we used the pRRophetic algorithm to estimate the chemotherapeutic response based on IC_{50} available in the GDSC database for each TCGA sample. The results indicated that the Immunity High subtype was more sensitive to the chemotherapies than the other two subtypes. Then, we used CMap based on differentially expressed genes, and found candidate compounds for possible differentiation therapy of LUAD patients. Moreover, we found that the Immunity High subtype had lower stemness index values than the other two subtypes; higher values for stemness indices signal higher biological activity in cancer stem cells and greater tumor dedifferentiation.[37] The above results implicate that the better prognosis with the Immunity High subtype may be because of a higher immunoreactive environment and because it inhibits tumor growth, progression, invasion, and metastasis. In addition, the Immunity High subtype may benefit more from immunotherapy and chemotherapy determined by these differences.

Our research provides new insights into the LUAD immune microenvironment. However, our research was limited because it was retrospective, and our results should thus be further confirmed by prospective studies. Additionally, the TCGA data enrolled for analysis were mostly collected from patients in developed countries but lacked data from developing countries.

Overall, for the first time, our study may provide a better assessment of the immune signature-based classification of LUAD. Our findings also infer potential treatments for the development of immunotherapeutic and chemotherapeutic strategies, and may guide the development of novel drug strategies.

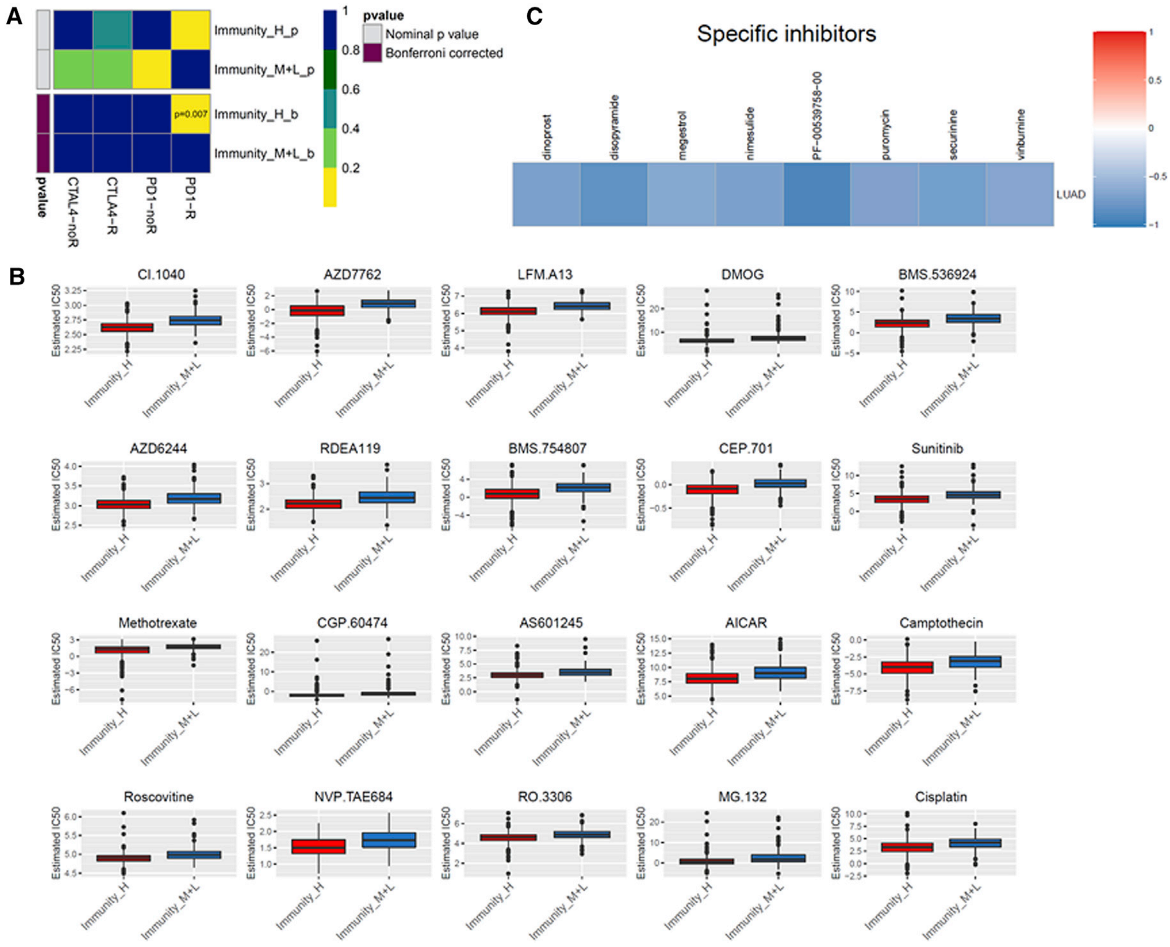

Figure 7. Evaluating the Therapeutic Response of the LUAD Subtypes

(A) Differential immunotherapeutic response targeting CTLA-4 and PD-1 in LUAD subtypes. (B) Differential chemotherapeutic response based on IC_{50} available in the GDSC database in LUAD subtypes. (C) Heatmap showing enrichment score of each compound from the CMap. IC_{50}, half-maximal inhibitory concentration.

MATERIALS AND METHODS

Data Source

Gene expression data and the corresponding clinical features for LUAD patients were accessed from TCGA website. This study meets TCGA's publication guidelines. All of the LUAD gene expression and clinical data were downloaded as determined by the Data Coordinating Center (DCC).

Hierarchical Clustering of LUAD Patients

To quantify the proportions of immune signatures in the LUAD samples based on the ssGSEA score, we used the 29 immune signatures, including cell types, functions, and pathways.[9]

Evaluation of Immune Microenvironment

Immune score and stromal score were evaluated by applying the ESTIMATE algorithm to the gene expression data from TCGA.[38,39] Tumor purity was obtained based on the ESTIMATE score using a fitted formula as previously described.[39]

Screening of Differentially Expressed Genes

The raw counts of TCGA gene expression were normalized and determined by a weighted trimmed mean of log ratios-based method.[40] In order to obtain differentially expressed genes, R package "limma" using the standard comparison mode was performed.[41] The threshold was determined as $|\log2 \text{ fold change (log2FC)}| \geq 1$ and false discovery rate (FDR) < 0.05.

Functional and Pathway Enrichment Analysis

Gene Ontology (GO) and the Kyoto Encyclopedia of Genes and Genomes (KEGG) pathway analysis using the clusterProfiler R package were performed on differentially expressed genes.[42] The thresholds for analyses were determined by a p value <0.05, indicating significantly enriched functional annotations.

Estimation of Tumor-Infiltrating Immune Cells

We uploaded the normalized gene expression data with standard annotation files to the CIBERSORT web portal, and the algorithm

was determined by 1,000 permutations and by the LM22 gene signature as described in previous literature.[43,44] The R “Genefilter” package was applied to screen each sample, and the threshold was determined as p value <0.05.

Immunotherapeutic and Chemotherapeutic Response Prediction

The PD-1/PD-L1 and CTLA-4 pathways in cancer are implicated in tumors escaping immune destruction; thus, immune checkpoint inhibitors targeting PD-1 and CTLA-4 enhance antitumor immunity.[45] Here, in order to predict the clinical response to immune checkpoint inhibitors, we ran the TIDE algorithm and subclass mapping as described previously.[46] Considering that chemotherapy is a common clinical practice to treat NSCLC, we applied the R package pRRophetic to estimate the chemotherapeutic response determined by the IC_{50} for each LUAD patient on the GDSC website.[47,48]

Calculation of Stemness Index

Stemness indices were calculated using an innovative OCLR machine-learning algorithm as previously described.[14,38] Then, we calculated Spearman correlations between the stemness index model and the lung cancer sample’s expression profile from TCGA. The stemness indices were subsequently mapped to the [0,1] range via utilizing a linear transformation that subtracted the minimum and divided by the maximum.

Compounds Therapeutic Response Prediction

To identify which target compounds might be useful, we used the CMap in predicting which compounds based on the top 1,000 differentially expressed genes.[14]

Statistical Analysis

All statistical analyses were performed using R version 3.6.1, and the data from different groups were compared by Mann-Whitney-Wilcoxon test. Pearson’s chi-square test was performed to measure the level of significance for association among variables. All reported p values were two-tailed, and $p < 0.05$ was considered statistically significant.

SUPPLEMENTAL INFORMATION

Supplemental Information can be found online at https://doi.org/10.1016/j.omto.2020.03.021.

AUTHOR CONTRIBUTIONS

F.X. and L.L. designed the study, analyzed data, and wrote the manuscript. J.-x.C., X.-b.Y., X.-b.H., and Z.-x.L. analyzed data and contributed in writing the manuscript. Y.-s.C. supervised the research, analyzed data, and wrote the manuscript.

CONFLICTS OF INTEREST

The authors declare no competing interests.

ACKNOWLEDGMENTS

This study was supported by grants from the National Natural Science Foundation of China (81672640); the Grant for Key Disciplinary Project of Clinical Medicine under the Guangdong High-level University Development Program (002-18119101); the Project of Innovating and Strengthening Universities in Guangdong Province supported by Department of Education of Guangdong Province (2018KTSCX066); the Special Funds for Innovation Strategy of Science and Education in Guangdong Province (2018-157); the Special Funds for Science and Technology of Guangdong Province (2019-113); the Science and Technology Planning Project of Shantou City (2019-77 and 2019-106); the Supporting Program of the First Affiliated Hospital of Shantou University Medical College (2019-70); the Guangdong Basic and Applied Basic Research Foundation (2020A1515011519); and the Medical Science and Technology Research Foundation of Guangdong Province (A2020430).

REFERENCES

1. Wan, L., Zhang, L., Fan, K., Cheng, Z.X., Sun, Q.C., and Wang, J.J. (2016). Knockdown of Long Noncoding RNA PCAT6 Inhibits Proliferation and Invasion in Lung Cancer Cells. Oncol. Res. *24*, 161–170.
2. Barata, A.T., Santos, C., Cravo, M., Vinhas, M.D., Morais, C., Carolino, E., Mendes, L., Roldão Vieira, J., and Fonseca, J. (2017). Handgrip Dynamometry and Patient-Generated Subjective Global Assessment in Patients with Nonresectable Lung Cancer. Nutr. Cancer *69*, 154–158.
3. Mony, J.T., and Schuchert, M.J. (2018). Prognostic Implications of Heterogeneity in Intra-tumoral Immune Composition for Recurrence in Early Stage Lung Cancer. Front. Immunol. *9*, 2298.
4. Liu, X., Wu, S., Yang, Y., Zhao, M., Zhu, G., and Hou, Z. (2017). The prognostic landscape of tumor-infiltrating immune cell and immunomodulators in lung cancer. Biomed. Pharmacother. *95*, 55–61.
5. Johnson, S.K., Kerr, K.M., Chapman, A.D., Kennedy, M.M., King, G., Cockburn, J.S., and Jeffrey, R.R. (2000). Immune cell infiltrates and prognosis in primary carcinoma of the lung. Lung Cancer *27*, 27–35.
6. Nadal, E., Massuti, B., Dómine, M., García-Campelo, R., Cobo, M., and Felip, E. (2019). Immunotherapy with checkpoint inhibitors in non-small cell lung cancer: insights from long-term survivors. Cancer Immunol. Immunother. *68*, 341–352.
7. Shukuya, T., and Carbone, D.P. (2016). Predictive Markers for the Efficacy of Anti-PD-1/PD-L1 Antibodies in Lung Cancer. J. Thorac. Oncol. *11*, 976–988.
8. Chen, L., and Han, X. (2015). Anti-PD-1/PD-L1 therapy of human cancer: past, present, and future. J. Clin. Invest. *125*, 3384–3391.
9. He, Y., Jiang, Z., Chen, C., and Wang, X. (2018). Classification of triple-negative breast cancers based on Immunogenomic profiling. J. Exp. Clin. Cancer Res. *37*, 327.
10. Givechian, K.B., Garner, C., Benz, S., Song, B., Rabizadeh, S., and Soon-Shiong, P. (2019). An immunogenic NSCLC microenvironment is associated with favorable survival in lung adenocarcinoma. Oncotarget *10*, 1840–1849.
11. Song, Q., Shang, J., Yang, Z., Zhang, L., Zhang, C., Chen, J., and Wu, X. (2019). Identification of an immune signature predicting prognosis risk of patients in lung adenocarcinoma. J. Transl. Med. *17*, 70.
12. Shi, X., Li, R., Dong, X., Chen, A.M., Liu, X., Lu, D., Feng, S., Wang, H., and Cai, K. (2020). IRGS: an immune-related gene classifier for lung adenocarcinoma prognosis. J. Transl. Med. *18*, 55.
13. Zhang, L., Zhang, Z., and Yu, Z. (2019). Identification of a novel glycolysis-related gene signature for predicting metastasis and survival in patients with lung adenocarcinoma. J. Transl. Med. *17*, 423.
14. Malta, T.M., Sokolov, A., Gentles, A.J., Burzykowski, T., Poisson, L., Weinstein, J.N., Kamińska, B., Huelsken, J., Omberg, L., Gevaert, O., et al.; Cancer Genome Atlas Research Network (2018). Machine Learning Identifies Stemness Features Associated with Oncogenic Dedifferentiation. Cell *173*, 338–354.e15.

15. Lin, J.H. (2016). Review structure- and dynamics-based computational design of anticancer drugs. Biopolymers *105*, 2–9.

16. Roberts, M.J., Broome, R.E., Kent, T.C., Charlton, S.J., and Rosethorne, E.M. (2018). The inhibition of human lung fibroblast proliferation and differentiation by Gs-coupled receptors is not predicted by the magnitude of cAMP response. Respir. Res. *19*, 56.

17. Liu, Z., Zhang, R., Chen, X., Yao, P., Yan, T., Liu, W., Yao, J., Sokhatskii, A., Gareev, I., and Zhao, S. (2019). Identification of hub genes and small-molecule compounds related to intracerebral hemorrhage with bioinformatics analysis. PeerJ *7*, e7782.

18. Pérez-Ramírez, C., Cañadas-Garre, M., Robles, A.I., Molina, M.A., Faus-Dáder, M.J., and Calleja-Hernández, M.A. (2016). Liquid biopsy in early stage lung cancer. Transl. Lung Cancer Res. *5*, 517–524.

19. Cao, B., Dai, W., Ma, S., Wang, Q., Lan, M., Luo, H., Chen, T., Yang, X., Zhu, G., Li, Q., and Lang, J. (2019). An EV-Associated Gene Signature Correlates with Hypoxic Microenvironment and Predicts Recurrence in Lung Adenocarcinoma. Mol. Ther. Nucleic Acids *17*, 879–890.

20. Wang, Y., Zhang, Q., Gao, Z., Xin, S., Zhao, Y., Zhang, K., Shi, R., and Bao, X. (2019). A novel 4-gene signature for overall survival prediction in lung adenocarcinoma patients with lymph node metastasis. Cancer Cell Int. *19*, 100.

21. Liu, C., Li, Y., Wei, M., Zhao, L., Yu, Y., and Li, G. (2019). Identification of a novel glycolysis-related gene signature that can predict the survival of patients with lung adenocarcinoma. Cell Cycle *18*, 568–579.

22. Li, S., Xuan, Y., Gao, B., Sun, X., Miao, S., Lu, T., Wang, Y., and Jiao, W. (2018). Identification of an eight-gene prognostic signature for lung adenocarcinoma. Cancer Manag. Res. *10*, 3383–3392.

23. Xia, L., Liu, Y., and Wang, Y. (2019). PD-1/PD-L1 Blockade Therapy in Advanced Non-Small-Cell Lung Cancer: Current Status and Future Directions. Oncologist *24* (*Suppl 1*), S31–S41.

24. Darvin, P., Toor, S.M., Sasidharan Nair, V., and Elkord, E. (2018). Immune checkpoint inhibitors: recent progress and potential biomarkers. Exp. Mol. Med. *50*, 1–11.

25. Chowell, D., Morris, L.G.T., Grigg, C.M., Weber, J.K., Samstein, R.M., Makarov, V., Kuo, F., Kendall, S.M., Requena, D., Riaz, N., et al. (2018). Patient HLA class I genotype influences cancer response to checkpoint blockade immunotherapy. Science *359*, 582–587.

26. Ribas, A., and Hu-Lieskovan, S. (2016). What does PD-L1 positive or negative mean? J. Exp. Med. *213*, 2835–2840.

27. Desrosiers, R., Friderici, K., and Rottman, F. (1974). Identification of methylated nucleosides in messenger RNA from Novikoff hepatoma cells. Proc. Natl. Acad. Sci. USA *71*, 3971–3975.

28. Chai, R.C., Wu, F., Wang, Q.X., Zhang, S., Zhang, K.N., Liu, Y.Q., Zhao, Z., Jiang, T., Wang, Y.Z., and Kang, C.S. (2019). m^6A RNA methylation regulators contribute to malignant progression and have clinical prognostic impact in gliomas. Aging (Albany NY) *11*, 1204–1225.

29. Yang, S., Wei, J., Cui, Y.H., Park, G., Shah, P., Deng, Y., Aplin, A.E., Lu, Z., Hwang, S., He, C., and He, Y.Y. (2019). m^6A mRNA demethylase FTO regulates melanoma tumorigenicity and response to anti-PD-1 blockade. Nat. Commun. *10*, 2782.

30. Sasidharan Nair, V., and Elkord, E. (2018). Immune checkpoint inhibitors in cancer therapy: a focus on T-regulatory cells. Immunol. Cell Biol. *96*, 21–33.

31. Long, L., Zhao, C., Ozarina, M., Zhao, X., Yang, J., and Chen, H. (2019). Targeting Immune Checkpoints in Lung Cancer: Current Landscape and Future Prospects. Clin. Drug Investig. *39*, 341–353.

32. Taniguchi, T., Ogasawara, K., Takaoka, A., and Tanaka, N. (2001). IRF family of transcription factors as regulators of host defense. Annu. Rev. Immunol. *19*, 623–655.

33. Murtas, D., Maric, D., De Giorgi, V., Reinboth, J., Worschech, A., Fetsch, P., Filie, A., Ascierto, M.L., Bedognetti, D., Liu, Q., et al. (2013). IRF-1 responsiveness to IFN-γ predicts different cancer immune phenotypes. Br. J. Cancer *109*, 76–82.

34. Nam, S., and Lim, J.S. (2016). Essential role of interferon regulatory factor 4 (IRF4) in immune cell development. Arch. Pharm. Res. *39*, 1548–1555.

35. Niu, J., Jiang, C., Li, C., Liu, L., Li, K., Jian, Z., and Gao, T. (2011). Foxp3 expression in melanoma cells as a possible mechanism of resistance to immune destruction. Cancer Immunol. Immunother. *60*, 1109–1118.

36. Yan, M., Himoudi, N., Pule, M., Sebire, N., Poon, E., Blair, A., Williams, O., and Anderson, J. (2008). Development of cellular immune responses against PAX5, a novel target for cancer immunotherapy. Cancer Res. *68*, 8058–8065.

37. Lian, H., Han, Y.P., Zhang, Y.C., Zhao, Y., Yan, S., Li, Q.F., Wang, B.C., Wang, J.J., Meng, W., Yang, J., et al. (2019). Integrative analysis of gene expression and DNA methylation through one-class logistic regression machine learning identifies stemness features in medulloblastoma. Mol. Oncol. *13*, 2227–2245.

38. Daily, K., Ho Sui, S.J., Schriml, L.M., Dexheimer, P.J., Salomonis, N., Schroll, R., Bush, S., Keddache, M., Mayhew, C., Lotia, S., et al. (2017). Molecular, phenotypic, and sample-associated data to describe pluripotent stem cell lines and derivatives. Sci. Data *4*, 170030.

39. Yoshihara, K., Shahmoradgoli, M., Martínez, E., Vegesna, R., Kim, H., Torres-Garcia, W., Treviño, V., Shen, H., Laird, P.W., Levine, D.A., et al. (2013). Inferring tumour purity and stromal and immune cell admixture from expression data. Nat. Commun. *4*, 2612.

40. Robinson, M.D., and Oshlack, A. (2010). A scaling normalization method for differential expression analysis of RNA-seq data. Genome Biol. *11*, R25.

41. Robinson, M.D., McCarthy, D.J., and Smyth, G.K. (2010). edgeR: a Bioconductor package for differential expression analysis of digital gene expression data. Bioinformatics *26*, 139–140.

42. Yu, G., Wang, L.G., Han, Y., and He, Q.Y. (2012). clusterProfiler: an R package for comparing biological themes among gene clusters. OMICS *16*, 284–287.

43. Zhou, R., Zhang, J., Zeng, D., Sun, H., Rong, X., Shi, M., Bin, J., Liao, Y., and Liao, W. (2019). Immune cell infiltration as a biomarker for the diagnosis and prognosis of stage I-III colon cancer. Cancer Immunol. Immunother. *68*, 433–442.

44. Xu, F., Zhang, H., Chen, J., Lin, L., and Chen, Y. (2020). Immune signature of T follicular helper cells predicts clinical prognostic and therapeutic impact in lung squamous cell carcinoma. Int. Immunopharmacol. *81*, 105932.

45. Postow, M.A., Callahan, M.K., and Wolchok, J.D. (2015). Immune Checkpoint Blockade in Cancer Therapy. J. Clin. Oncol. *33*, 1974–1982.

46. Lu, X., Jiang, L., Zhang, L., Zhu, Y., Hu, W., Wang, J., Ruan, X., Xu, Z., Meng, X., Gao, J., et al. (2019). Immune Signature-Based Subtypes of Cervical Squamous Cell Carcinoma Tightly Associated with Human Papillomavirus Type 16 Expression, Molecular Features, and Clinical Outcome. Neoplasia *21*, 591–601.

47. Geeleher, P., Cox, N.J., and Huang, R.S. (2014). Clinical drug response can be predicted using baseline gene expression levels and in vitro drug sensitivity in cell lines. Genome Biol. *15*, R47.

48. Xu, F., Lin, H., He, P., He, L., Chen, J., Lin, L., and Chen, Y. (2020). A *TP53*-associated gene signature for prediction of prognosis and therapeutic responses in lung squamous cell carcinoma. OncoImmunology *9*, 1731943.

OMTO, Volume *17*

Supplemental Information

Analysis of Lung Adenocarcinoma Subtypes Based on Immune Signatures Identifies Clinical Implications for Cancer Therapy

Feng Xu, Jie-xin Chen, Xiong-bin Yang, Xin-bin Hong, Zi-xiong Li, Ling Lin, and Yong-song Chen

Supplemental Figure 1 Hierarchical clustering of LUAD patients.

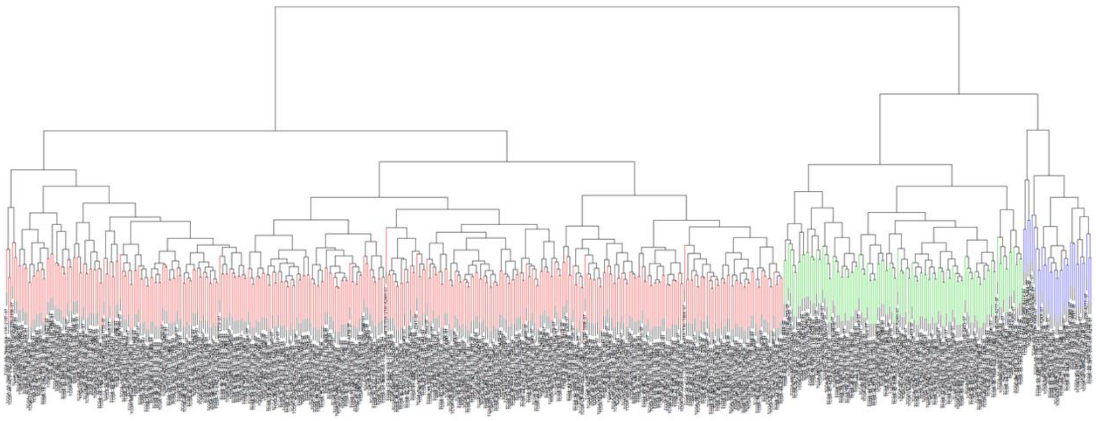

Supplemental Figure 2 Stemness indices in LUAD subtypes.

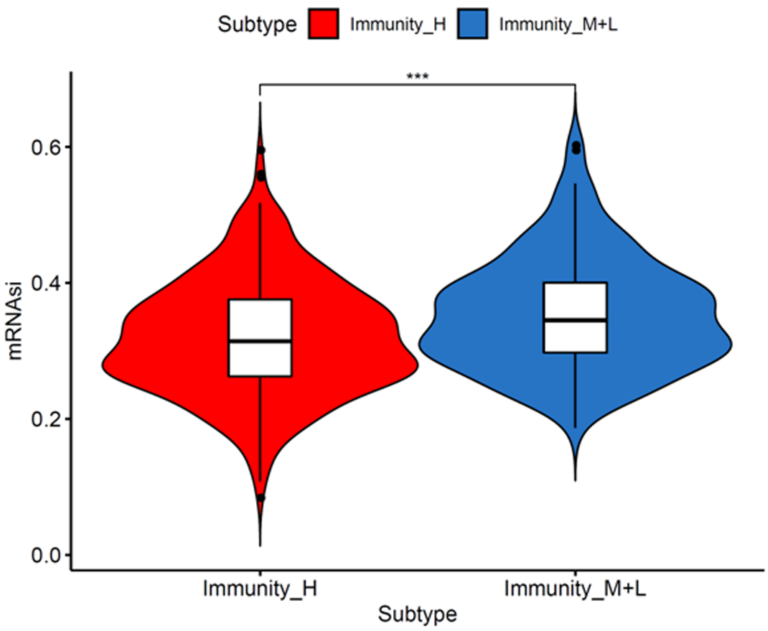

Supplemental Figure 3 Relationship between the immune signature model and the clinical information. (A) Nomogram for predicting the probability of 1-, 3-, and 5-year overall survival for LUAD patients.; (B) Time-dependent ROC curve analyses of the immune signature model, gender, TNM stage, T stage, N stage, M stage.

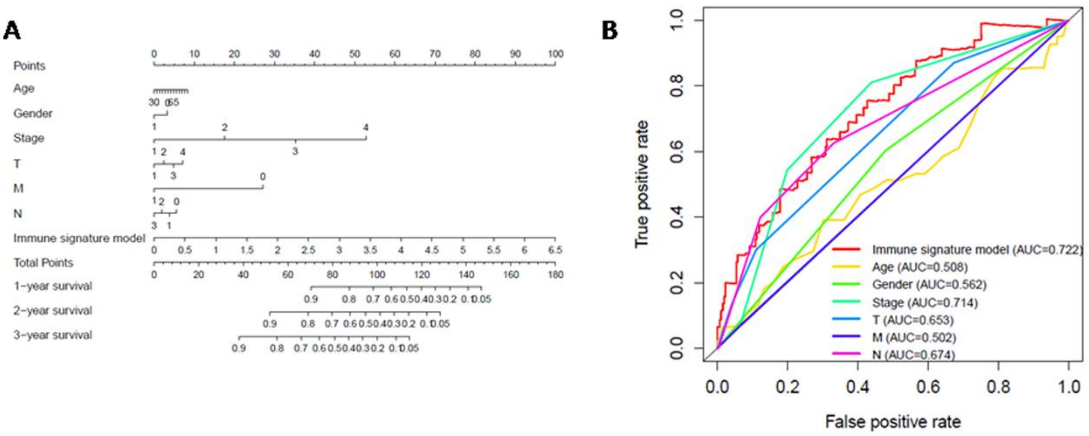

Supplemental table 1 chemotherapeutic response

GDSC name	p	Synonyms	Targets	Target way
CI.1040	2.04E-23	CI 1040, PD-18435, PD-184352, 212631-79-3	MEK1, MEK2	ERK MAPK signaling
AZD7762	4.84E-23	AZD-7762, AZD 7762	CHEK1, CHEK2	Cell cycle
LFM.A13	3.93E-22	DDE-28	BTK	Other, kinases
DMOG	4.53E-20	Dimethyloxalylglcine	HIF-PH	Metabolism
BMS.536924	1.22E-18	BMS 536924	IGF1R, IR	IGF1R signaling
AZD6244	1.98E-18	AZD6244, AZD-6244, ARRY-886，Selumetinib	MEK1, MEK2	ERK MAPK signaling
RDEA119	2.01E-18	RDEA119, BAY-86-9766, BAY 869766	MEK1, MEK2	ERK MAPK signaling
BMS.754807	2.17E-17	BMS754807, BMS 754807	IGF1R, IR	RTK signaling
CEP.701	2.55E-17	CEP-701, SP-924, SPM-924, A-154475, KT-555，Lestaurtinib	FLT3, JAK2, NTRK1, NTRK2, NTRK3	Other, kinases
Sunitinib	3.45E-17	Sutent, Sunitinib Malate, SU-11248	PDGFR, KIT, VEGFR, FLT3, RET, CSF1R	RTK signaling
Methotrexate	4.41E-15	Abitrexate, Amethopterin, Rheumatrex, Trexall, Folex	Antimetabolite	DNA replication
CGP.60474	6.33E-15	KIN001-019, CGP60474, CGP 60474	CDK1,CDK2,CDK5,CDK7,CDK9, PKC	Cell cycle
AS601245	2.89E-13	/	JNK1, JNK2, JNK2	JNK and p38 signaling
AICAR	3.81E-13	AICAR, N1-(b-D-Ribofuranosyl)-5-aminoimidazole-4-carboxamide	AMPK agonist	Metabolism
Camptothecin	8.97E-13	Camptothecine, (+)-Camptothecin	TOP1	DNA replication
Roscovitine	1.69E-12	Roscovitine, CYC-202, AL-39256	CDK2, CDK7, CDK9	Cell cycle
NVP.TAE684	2.05E-12	NVP-TAE 684, TAE684, TAE-684	ALK	RTK signaling
RO.3306	7.05E-12	/	CDK1	Cell cycle
Cisplatin	1.27E-11	cis-Diammineplatinum(II) dichloride, Platinol, CIS-DDP	DNA crosslinker	DNA replication
MG.132	1.27E-11	LLL cpd, MG 132, MG132	Proteasome, CAPN1	Protein stability and degradation
